# Dermato-cosmeceutical properties of *Pseudobombax ellipticum* (Kunth) Dugand: Chemical profiling, *in vitro* and *in silico* studies

**DOI:** 10.1016/j.jsps.2023.101778

**Published:** 2023-09-01

**Authors:** Eman Fikry, Ismail Mahdi, Ahmet Buğra Ortaakarsu, Nora Tawfeek, Melvin Adhiambo Ochieng, Widad Ben Bakrim, Mohamed AO Abdelfattah, Khaled W. Omari, Mona F. Mahmoud, Mansour Sobeh

**Affiliations:** a Department of Pharmacognosy, Faculty of Pharmacy, Zagazig University, Zagazig 44519, Egypt; b AgroBioSciences Program, College for Sustainable Agriculture and Environmental Science, Mohammed VI Polytechnic University, Ben Guerir 43150, Morocco; c Department of Chemistry, Faculty of Science, Gazi University, Turkey; d College of Engineering and Technology, American University of the Middle East, Kuwait; e Department of Pharmacology and Toxicology, Faculty of Pharmacy, Zagazig University, Zagazig 44519, Egypt

**Keywords:** Antiaging, Biofilm inhibition, Docking, *Pseudobombax ellipticum*

## Abstract

Plant extracts and their individual components have been used to manage skin aging for several decades. Recently, the discovery of new natural bioactive agents, that not only enhance the skin health but also offer protection against various deleterious factors, such as free radicals, ultraviolet radiation, and microbial infections, has been a potential target by many researchers. The aim of the current work was to investigate the phytochemical profile of an ethanol bark extract from *Pseudobombax ellipticum*, and to evaluate its antioxidant, antiaging and antibacterial activities *in vitro*. Molecular docking and molecular dynamics studies were adopted to estimate and confirm the binding affinity of several compounds and explain their binding pattern at the binding sites of four target enzymes associated with skin aging, namely collagenase, elastase, tyrosinase, and hyaluronidase. HPLC-MS/MS analysis led to the tentative identification of 35 compounds comprising phenolic acids, and their glycosides, procyanidins and flavonoid glycosides. The extract demonstrated a promising *in vitro* antioxidant activity in the DPPH and FRAP assays (IC_50_ 56.45 and 15.34 μg/mL, respectively), and was able to inhibit the aforementioned key enzymes with comparable results to the reference drugs. In addition, the extract (6.25 mg/mL) inhibited the biofilm production of *Pseudomonas aeruginosa* and diminished the swimming and swarming motilities. The docked compounds revealed appreciable binding energy with the tested enzymes and were stable throughout the molecular dynamic simulations. In view of this data, *P. ellipticum* bark can be regarded as a good candidate for prospective application in derma-cosmeceutical preparations.

## 1 Introduction

The skin is the largest organ in humans that is liable to age-related diseases. Skin not only has a crucial role as a defensive border for the internal organs against different influences but also acts as a functional organ. Nowadays, skin aging is a significant concern for scientists, leading to a significant surge in research on this topic. There is now a growing preference for using plant extracts in skincare products due to their ecological friendliness, gentle nature on the skin surface, and ability to be easily incorporated into daily routines with minimal side effects. Moreover, herbal skincare products offer various benefits such as antioxidants, antimicrobial properties, and the ability to inhibit pigmentation, making them suitable for different skin conditions (Emerald et al., 2016, Ahmed et al., 2020, Xie et al., 2022).

Skin diseases are prevalent among elderly individuals due to their weakened skin and disrupted skin barrier function, which is more pronounced compared to younger individuals. Numerous studies have been conducted to evaluate the epidemiology of skin diseases in the elderly population. Skin aging is characterized by histological, morphological, and physiological changes that occur as a result of aging. It can be classified as intrinsic or extrinsic, depending on the underlying epidemiological factors that contribute to the aging process. Intrinsic aging is primarily influenced by chronological and genetic factors, while extrinsic aging, also known as photoaging, is influenced by environmental factors (Hahnel et al., 2017, Wong and Chew, 2021).

Intrinsic aging is mainly induced by hormones, genetics, metabolism side products as reactive oxygen species (ROS) which are the most harmful factors. While extrinsic one results from exposure to extrinsic factors as UV radiation, air pollution, chemicals and other external environmental factors (George et al., 2022). To protect against these external environmental factors, the skin is formed from multiple layers to make proper defense against these harmful factors. These layers include epidermis, dermis, and hypodermis. The epidermis is the outermost layer of skin, made of four closely adherent layers and comprising keratinocytes, with different stages of differentiation, in which the protein keratin is synthesized. The epidermis acts as a biological and physical barrier against the outside environment, irritants, and allergens. In addition, it prevents water loss. The dermis is thicker than epidermis and its main functions are support, protection, and wound healing. It consists of two layers and its major cell types are fibroblasts which synthesize elastin, collagen, and the viscous gel within this layer. The skin’s bottom layer, hypodermis, is mainly responsible for energy storage and insulation (Lawton 2019).

Several studies revealed the relation between skin aging and oxidative stress (Lee et al., 2021). The latter is an imbalance between oxidative and anti-oxidative species and characterized by excessive free radical production in the skin, which is the main factor responsible for skin aging. Free radicals such as reactive oxygen species (ROS) as superoxide anion and hydroxyl radical are induced by both external and internal factors. External factors comprise exposure to chemicals, toxins, pathogens, and ultraviolet (UV) light. While internal factors include different inflammations and hormonal imbalances. The excessive ROS promotes an elevation in matrix metalloproteinases (MMPs) expression in humans’ skin. These MMPs, constituting a super family of protease enzymes, are responsible for the degradation of the skin extracellular matrix (ECM) fibrous proteins such as elastin, collagen and hyaluronic acid (Michalak 2022). The major signs of skin aging are appearance of wrinkles due to breakdown of these fibrous proteins by the ROS-induced collagenase, elastase, and hyaluronidase enzymes, respectively, hence associated with poor skin elasticity, and declined wound healing ability. Moreover, the increased production of melanin is another major indication for skin aging and is organized by the enzyme tyrosinase. Hence, targeting these mentioned enzymes using inhibitors is a promising mechanism in fighting against skin aging, notably if these inhibitors possess strong antioxidant potential (Younis et al., 2022).

*Pseudomonas aeruginosa* is a Gram-negative pathogen that is commonly associated with opportunistic skin infections, ranging from localized skin infections to systemic disease. This pathogen is known for its ability to form a polymeric matrix, or biofilm, which confers resistance to *P. aeruginosa* cells against various antibiotics and the host's immune system by impeding drug penetration through bacterial surfaces. The biofilm complex is composed of several components, including polysaccharides, extracellular DNA, lipids, and proteins. Therefore, inhibiting biofilm formation may be a crucial strategy in combating the increasing bacterial resistance to antibiotics. As such, it is imperative to assess the antibiofilm activity of the extract under investigation (Spernovasilis et al., 2021).

Bombacoideae, which was formerly categorized as part of the Bombacaceae family, is now recognized as a subfamily of Malvaceae as per the recent molecular and phytochemical studies. These plants are a source of various phytoconstituents, including flavonoids, alkaloids, and tannins, and have significant economic and medicinal value (Das et al., 2021).

The *Pseudobombax* genus is part of the Bombacoideae family and consists of around 30 species found in the Neotropics. *Pseudobombax ellipticum* is a deciduous tree that grows up to 18–30 m tall and 1.3–1.5 m in diameter. It has a straight, succulent trunk with branches close to the base, palmately compound leaves with five leaflets, and solitary pink or white flowers with numerous stamens. The tree produces elongated capsules with numerous seeds. *P. ellipticum* can thrive in rocky, dry habitats or poor soils. It sheds its leaves from December to May, flowers in December or January, and matures its fruits in January and February. The tree is grown as an ornamental plant in Florida and Hawaii and is used to decorate homes and churches in Central America due to its attractive flowers. It can be propagated by seeds or cuttings (Ávila-Calderón and Rutiaga-Quiñones, 2015, Walker, 2021).

Medicinally, *P. ellipticum* has been reported to be effective in respiratory disorders such as cough, and against fever and as an antimicrobial drug (Ruiz-Terán et al., 2008). Additionally, the bark decoction has been used as a treatment for cough and catarrh in Guatemala (Das et al., 2021). In El Salvador, the tea of the flowers has been effective for gastrointestinal disorders while the tea of the fresh bark exhibited an antidiabetic effect (Vozzo 2002). Moreover, the aerial parts of *P. ellipticum* exhibited a significant radical scavenging and antioxidant activity (Ruiz-Terán et al., 2008). Besides, *β*-lupeol from the stem bark of *P. ellipticum* demonstrated a valuable gastroprotective activity (CHÁVEZ-PIÑA et al., 2009). Phytochemically, cyanidin-3,5-diglucoside has been identified as a constituent of the flowers of *P. ellipticum* (Das et al., 2021).

The literature survey clarified that no sufficient attempts have been submitted until now to explore the chemical composition and possible biological activities of *P. ellipticum* bark and leaves growing in Egypt. Moreover, it was found that some members of Malvaceae and their phenolic constituents can be used as antiaging agents through different mechanisms. In this regard, Hibiscus roseus flowers and leaves showed great antioxidant, sun-protection and anti-collagenase activities, making them ideal for inclusion in cosmetics (dos Santos Nascimento et al., 2021). Additionally, Hibiscus sabdariffa L. and hibiscus acid were also effective in delaying skin aging by promoting extracellular matrix synthesis in skin fibroblasts and exhibiting antioxidant capabilities (Wang et al., 2022). Based on this literature, there is a possibility that *P. ellipticum* extracts may contain beneficial active constituents for skin care and anti-aging products. Accordingly, the present work aimed to evaluate the antioxidant capacity and the *in vitro* inhibitory activity of the bark and leaves extracts against some protein key players in aging process including elastase, hyaluronidase, collagenase and tyrosinase enzymes. Furthermore, it was intended to investigate the effectiveness of the bark ethanol extract on biofilm production of the common pathogen in skin infections, *Pseudomonas aeruginosa*, and to profile the chemical constituents of this extract, using LC-MS/MS. In addition, the major compounds identified in this extract were docked to four enzymes associated with skin aging to study the possible molecular targets involved in the mechanism of action of the extract. Moreover, molecular dynamics were done to confirm the binding affinities of the docked compounds to the target proteins.

## 2 Material and methods

### 2.1 Plant material and extraction

*P. ellipticum* fresh bark and leaves were gathered (April 2021) from El-Orman Botanical Garden, Giza, Egypt. A voucher specimen for each was kept at the botanical herbarium of Pharmacognosy Department, Faculty of Pharmacy, Zagazig University with the accession number ZU-Ph-Cog-020103 and ZU-Ph-Cog-020104, respectively. The gathered bark was shadow-dried and ground, then ultrasound-assisted extraction (UAE) with water and 70% ethyl alcohol (10 g × 400 mL, each) for 15 min was carried out on the powdered bark. Then, filtration of aqueous and ethanol extracts was done with Glass-wool and centrifugation for 7 min at 6000 rpm. The collected filtrates were evaporated under reduced pressure by Buchi rotavapor R-300 (Flawil, Switzerland), then freeze dried to produce fine dried powders (1.18 g and 0.23 g, respectively). The leaves were extracted in a similar way to produce 1.93 g and 0.36 g for aqueous and ethanol extracts, respectively.

### 2.2 HPLC-PDA-MS/MS analysis

The phytochemical profile of *P. ellipticum* bark ethanolic extract was investigated using HPLC-PDA-MS/MS as previously reported (Tawfeek et al., 2023).

### 2.3 In silico studies

#### 2.3.1 Molecular docking

The molecular docking was done using the default protocol of the molecular operating environment software (MOE2022.v11.18.1) as previously described (Elgamal et al., 2021, Bogari et al., 2022). Detailed methods are provided in the supplementary file.

#### 2.3.2 Molecular dynamics

##### 2.3.2.1 Preparation of proteins

After the molecular docking calculation, the preparation of the proteins for the molecular dynamics’ calculation was started. The proteins obtained from the docking results (best poses) were in complex with their corresponding ligands. Each protein structure was prepared according to ionization states at physiological pH 7.4 using the Protein Preparation Wizard (L.L.C. Schrödinger, Schrödinger release 2023–1: Protein Preparation Wizard, Epic, Impact and Prime Schrödinger. LLC, New Y (2023)) available in Shrodinger Maestro (Schrödinger, Schrödinger Release 2023–1: Maestro, Schrödinger, LLC, Maestro-Desmond Interoperability Tools, Desmond Molecular Dynamics System. (2023)). Appropriate ionization states of the proteins and their ligands were adjusted using the Epic (Schrödinger, Epik | Schrödinger, Schrödinger Release 2023–1. (2023)) embedded software. Missing amino acid residues were added. Faulty hydrogens that do not make bonds in protein structures were added to amino acids appropriately. Finally, the protein structures were minimized using the OPLS3e force field (Roos, et al., 2019).

##### 2.3.2.2 System setup

Protein structures were prepared separately for each enzyme, with ligand (Halo Form) and without ligand (Apo Form) as it was aimed to examine the effects of ligands on the natural function of enzymes. In the apo form there is no ligand, in the halo form there is the ligand used in the calculation of molecular docking. Each protein construct was immersed in an orthorhombic solvent box measuring 10 Å × 10 Å × 10 Å. The water molecules in the solvent box were used in the SPC setting to achieve more realistic results (Mark and Nilsson, 2001, Gopal et al., 2015). Using the Monte Carlo method, system neutralization was achieved by adding sodium and chloride ions to the solvent box, 0.15 M NaCl was added as well in order to simulate physiological conditions. By selecting the force domain OPLS3 (Harder et al., 2016), the protein–ligand complex was prepared for molecular dynamics simulation (Table S1 in the supplementary file).

##### 2.3.2.3 Molecular dynamics simulations

The next step in evaluating the data from the molecular docking study was to initiate simulations of molecular dynamics, which involved studying the motions of atoms in their natural environment. For this purpose, the Desmond (N. D. E. Shaw Research, New York, Schrödinger Release 2023–1: Desmond Molecular Dynamics System, Maestro Desmond Interoperability Tools, Schrödinger, New York. (2023)) module in Maestro was used. Simulation of each protein structure was set to the NPT option to maintain a fixed number of particles, temperature, and pressure parameters. Besides, the simulations consisted of a 2 ps relaxation protocol. The crystal waters next to the protein structures have been preserved to obtain more realistic and experimental results. Nose-Hoover thermostat (Evans & Holian, 1985) is used to maintain the constant temperature in the systems and to ensure a proper simulation. By fixing the temperature in this system to 310 K, the simulations are enabled to take place at a constant temperature. The pressures of the systems were adjusted to 1 bar using a Martyna-Tobias-Klein barostat (Martyna et al., 1994). All these processes ultimately provided high-precision control over temperature and pressure and ensured that simulations were carried out accurately and reliably. A total of eight molecular dynamics simulations consisting of apoforms and haloforms were started at 100 ns each.

##### 2.3.2.4 Generalized born surface area (MM-GBSA)

The MM-PBSA calculation method provides numerical estimation of the interaction mechanisms of the complexes obtained from the molecular docking results and gives an idea about the stability of the complexes. It is very important to monitor how the obtained numerical data changes during the molecular dynamics simulation in order to view how the stability of the complexes changes over time. The MM-GBSA calculation was used to estimate the binding free energy of the complexes (Turkmen et al., 2022). MM-GBSA calculation was made once every 400 frames. The MM-GBSA calculation was performed using the OPLS-3e force field using the VSGB solvent model and the rotamer search algorithm with the Prime module in Maestro. The MM-GBSA calculation includes the values in the following equation:

ΔG = E complex (minimized) – [E ligand (minimized) + E receptor (minimized)]

The default setting, in which all the protein atoms were rendered rigid while those of ligands are relaxed, was utilized to calculate the MM-GBSA (Rastelli et al., 2010, Nada et al., 2022).

### 2.4 In vitro antioxidant assays

#### 2.4.1 DPPH and FRAP assays

The antioxidant activity was done using two colorimetric assays, DPPH(2,2-diphenyl-1-picrylhydrazyl) and FRAP (Ferric reducing antioxidant power) assays and the assays were done as previously reported (Ghareeb et al., 2018).

#### 2.4.2 *TPC and* TFC

The total polyphenolic compounds content (TPC) and total flavonoids content (TFC) were determined as previously described (Tawfeek et al., 2023).

### 2.5 In vitro enzymatic activities

#### 2.5.1 Collagenase inhibition

The assay was carried out as previously illustrated (Elgamal et al., 2021) with minor modifications. The assay was done in 50 mM Tricine buffer comprising 400 mM NaCl and 10 mM CaCl_2_, pH 7.5. Collagenase enzyme was produced from *Clostridium histolyticum* (ChC–EC.3.4.23.3) as an initial concentration of 0.8 unit/mL using the buffer as solvent. Also, tricine buffer was used as a solvent to yield 2 mM solution of the synthetic substrate FALGPA (N-[3-(2-furyl) acryloyl] -Leu-Gly-Pro-Ala). Serial diluted samples were prepared in concentrations 250–7.81 μg/ mL, incubated for 15 min with the enzyme in Tricine buffer. The enzyme reaction was started by adding the substrate to each serial solution. The absorbance (A) at 490 nm was monitored by a microplate reader and quercetin as positive control. Each measurement was performed in triplicate and the percentage of collagenase inhibition (%) was calculated by the formula: inhibition (*%*) = [(*A* control – *A* sample/*A* control) * 100].

#### 2.5.2 Tyrosinase inhibition

In this assay, L-DOPA was used as substrate and tyrosinase from mushroom as an enzyme following the previously reported procedure (Elgamal et al., 2021). The reaction included mushroom tyrosinase (2500 U mL^_1^, 15 μL), test samples (100 – 6.25 μg/mL, 15 μL), L-DOPA (5 mM, 100 μL), and phosphate buffer (0.05 M, 100 μL, pH of 6.5). The dopachrome formation in the reaction mixture was monitored at 475 nm using a Microplate reader, comparing it to Kojic acid as a positive control and performing each measurement in triplicate. Tyrosinase inhibition percentage (%) was measured by this formula: inhibition (*%*) = [(*A* control – *A* sample/*A* control) * 100].

#### 2.5.3 Elastase inhibition

This assay was performed following the previous study by (Elgamal et al., 2021). Sterile water was used to prepare a stock solution of Porcine pancreatic elastase enzyme (3.33 mg/mL), while buffer was used to prepare the substrate as 1.6 mM AAAPVN (N-Succinyl-Ala-Ala- Ala-pnitroanilide) solution. The incubation of serial diluted test samples (100 to 6.25 μg/mL) with the enzyme was conducted for 15 min, followed by the addition of AAAPVN substrate which initiated the enzyme reaction. So, a total volume of 250 μL of the final reaction mixture comprises 0.8 mM of AAAPVN, 1 μg/mL of PE, 25 μg of test sample, and the buffer vehicle. The absorbance (A) was observed at 400 nm with a microplate reader, positive control was kojic acid and each measurement was conducted in triplicate. The percentage of elastase enzyme inhibition (%) was calculated by the formula: inhibition (*%*) = [(*A* control – *A* sample/ *A* control) *100].

#### 2.5.4 Hyaluronidase inhibition

In this assay, 1.5 mg/mL of hyaluronidase enzyme and 1 mg/ml (in 0.1 M acetate buffer; pH 3.5) of the substrate hyaluronic acid was used as reported (Elgamal et al., 2021).The reaction mixture which consisted of 25 μL CaCl_2_ (12.5 mM), 12.5 μL of each tested sample(100 – 6.25 μg/mL), 1.5 mg/mL hyaluronidase enzyme, and 100 μL substrate hyaluronic acid were mixed in 2 mL-test tube. Then, adding 25 μL of KBO_2_ (0.8 M) to all tubes after heating the reaction mixture at 100 °C in a water bath for 3 min, cooling the tubes at the room temperature, adding 800 μL DMAB (4 g DMAB in 40 mL acetic acid and 5 mL 10 N HCl) to the tubes, incubation for 20 min, then finally, transferring the contents of the tubes to respective wells in a 96-well microplate. The absorbance (A) was observed at 600 nm and each measurement was done out in triplicate. The percentage of enzyme inhibition (%) was calculated by the formula: inhibition (*%*) = [(*A* control – *A* sample/*A* control) *100].

### 2.6 Antibacterial activities

#### 2.6.1 Determination of the minimum inhibitory concentration (MIC)

The broth microdilution assay (Abbas et al., 2017, Bakrim et al., 2022) using a 96-well microtiter microplate was used to investigate the MIC of *P. ellipticum* bark ethanol extract against *Pseudomonas aeruginosa*. Firstly, a final extract concentration of 100 mg/mL was obtained by dissolving the extract in Mueller-Hinton (MH) broth with 5% DMSO (dimethyl sulfoxide). Then, 0.22 μm sterile syringe filters were used to sterilize the extract, which was tow fold serially diluted into the plate’s wells in triplicate, yielding final extract concentrations range (1.562 to 100 mg/mL). The volume was completed to200 μL per well using MH medium, then bacterial suspensions of *P. aeruginosa* (fresh overnight, with turbidity of OD_600nm_ = 0.6) were mixed into each well (2 μL per well). Negative controls were wells without bacteria, positive control was ampicillin (10–0.078125 mg/mL), and growth controls were media without extract amendment. The plate was incubated at 37 °C and shaked at 150 rpm for 18 h. The bacterial growth was observed and spectrophotometrically evaluated.

#### 2.6.2 Biofilm inhibition using crystal violet assay

The crystal violet colorimetric assay (Mostafa et al., 2020, Mahdi et al., 2021) using a 96-well microtiter microplate was used to determine the influence of the sub-MIC concentrations of *P. ellipticum* bark ethanol extract on biofilm production by *P. aeruginosa*. First, the extract (doses of 1/8 MIC and 1/4 MIC) was prepared using MH broth, filtered with 0.22 μm syringe filters, inoculated, then incubated as mentioned above. After 18 h, the bacterial suspensions were thrown away followed by removal of the nonadherent bacterial cells through washing the wells twice with PBS (phosphate-buffered saline). Then, 1% CV (crystal violet) solution (200 μL per well) was used to stain adherent bacteria, incubated at room temperature. Fifteen minutes later, CV solutions were removed, and sterile distilled water was used to remove excess dye through vigorous washing of the wells. The plate was then dried by air, solubilizing the attached biofilm by adding 95% ethanol (200 μL) into each well. Multimode plate reader was used to estimate the amount of biofilm in each media spectrophotometrically at OD_600._ The positive controls for biofilm production were media without the extract.

#### 2.6.3 Swimming and swarming mobilities assessment on plates

*P. ellipticum* bark ethanol extract at 1/4 and 1/8 sub-MIC concentrations was assessed on the swimming and swarming motilities of *P. aeruginosa* (Mostafa et al., 2020). The swimming plates are consisting of 1% tryptone, 0.5% sodium chloride, and 0.3% agar, while swarming plates are consisting of semisolid LB medium and 0.6% agar (Yeung et al., 2009). The plates were prepared and autoclaved. After cooling the media (<50 °C), they were supplied with the filtered extract yielding final doses of 1/8 and 1/4 MIC followed by drying the plates under a laminar flow hood. subsequently, a fresh suspension of *P. aeruginosa* (10 μL, OD_600 nm_ = 0.6) was kept at the center of each agar plate, then incubated for 24 h at 37 °C. The controls were media without extracts. The swimming and swarming zone diameters were calculated in cm.

### 2.7 Statistical analysis

Each experiment was performed in triplicate throughout the study, and the results were calculated as means ± standard deviation (SD). The significant differences between the group means were calculated through the Tukey's post hoc test using IBM SPSS software. It was considered statistically significant when P < 0.05.

## 3 Results

### 3.1 Phytochemical profiling by HPLC-PDA-MS/MS

**Fig. 1:**
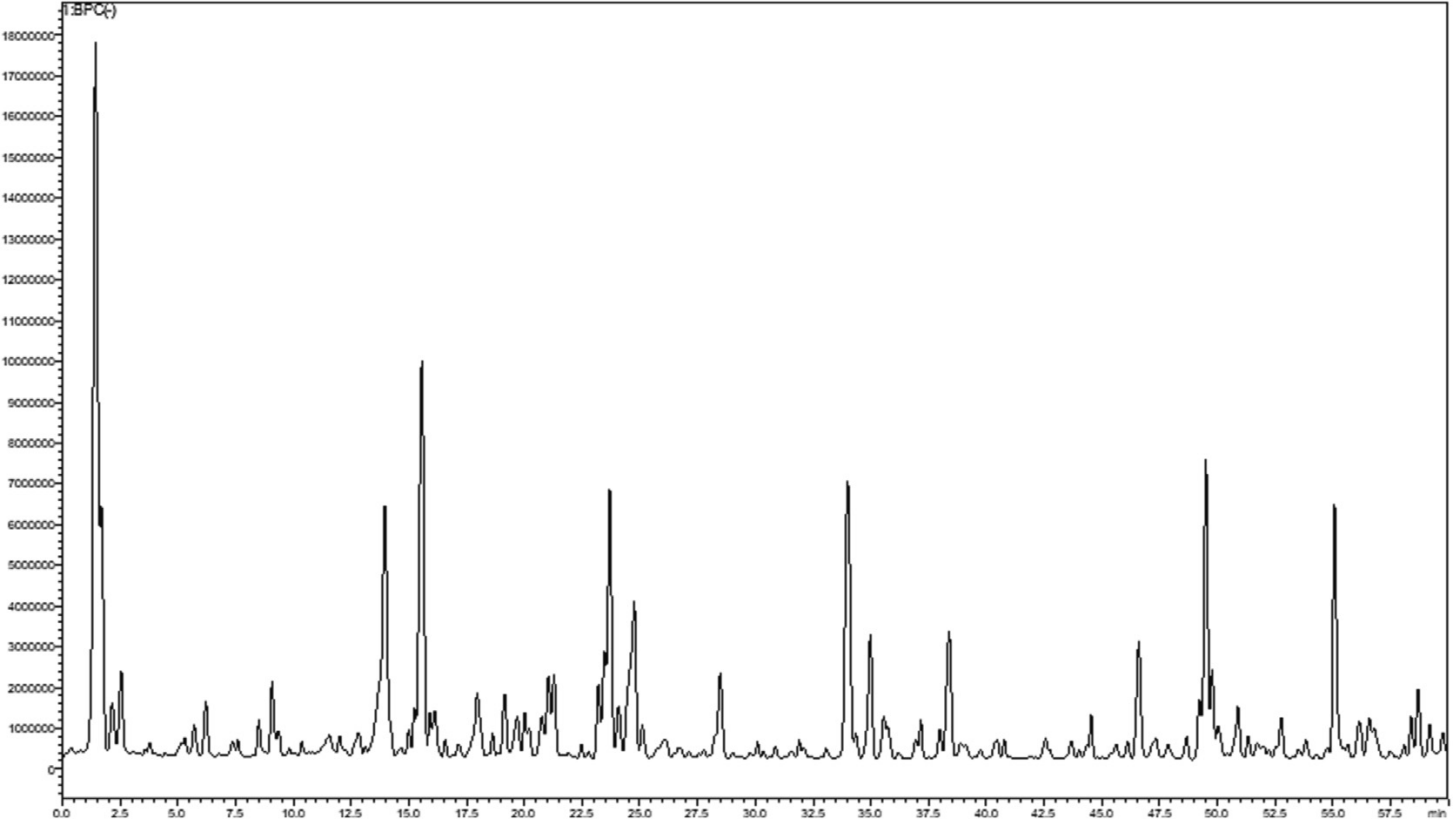
LC-MS profile of *P. ellipticum* bark extract.

In total, 35 compounds from various chemical classes were characterized through HPLC-PDA-MS/MS predominating the ethanol extract, Fig. 1. The compounds were tentatively identified using their MS, MS^2^ fragments and retention times as presented in Table 1. The identified compounds included organic and phenolic acids such as malic and quinic acids (free and their conjugates), catechins, flavonoid aglycones and glycosides such as quercetin and its glycosides (mono, diglucosides and rutin).

**Table 1:** Identified metabolites by HPLC-MS/MS analyses of the ethanolic extract of *P. ellipticum* bark.

**No.**	**Rt (min)**	**[M−H]^-^**	**MS/MS**	**Annotated metabolites**
	1.44	191	109	Quinic acid
	1.64	133		Malic acid
	2.59	279	163	Coumaroyl malic acid
	4.88	315	152	Protocatechuic acid glucoside
	6.08	153	108	Protocatechuic acid
	7.05	353	191	Chlorogenic acid
	8.48	339	133	Sinapoyl malate
	9.99	337	191	Coumaroylquinic acid
	11.14	289	173	Shikimic acid malate
	11.25	325	163	Coumaroyl glucose
	11.32	369	193	Ferulic acid glucuronide
	11.68	357	119	Dihydroferulic acid glucoside
	12.09	353	191	Chlorogenic acid
	12.64	387	179	Dihydrosinapic acid glucoside
	13.05	577	289	(epi)-Catechin-(epi)-catechin
	13.24	865	289	(epi)-Catechin-(epi)-catechin-(epi)-catechin
	13.66	355	179	Ferulic acid glucose
	13.96	593	305	(epi)-Gallocatechin-(epi)-catechin
	15.34	289	205	Epicatechin
	15.94	421	289	Catechin pentoside
	16.07	457	289	Epigallocatechin gallate
	17.32	865	289	(epi)-Catechin-(epi)-catechin-(epi)-catechin
	18.76	625	301	Quercetin diglucoside
	19.79	865	289	Procyanidin trimer
	23.09	609	301	Rutin
	24.00	463	301	Quercetin glucoside
	24.36	577	289	(epi)-Catechin-(epi)-catechin
	24.78	865	289	(epi)-Catechin-(epi)-catechin-(epi)-catechin
	27.18	593	285	Kaempferol rutinoside
	28.45	187		Ethyl gallate
	33.80	609	315	Isorhamnetin pentosyl-glucoside
	34.76	623	315	Isorhamnetin rutinoside
	37.94	579	315	Isorhamnetin dipentoside
	38.37	625	331	Methylmyricetin pentosyl-glucoside
	38.97	301	179	Quercetin

### 3.2 In silico results

#### 3.2.1 Molecular docking

Herein, an *in silico* molecular docking was performed to obtain a clear knowledge about the binding affinity and the binding modes of the extract’s compounds at the binding sites of the target elastase, tyrosinase, collagenase, and hyaluronidase enzymes. In this study, nine major secondary metabolites identified in *P. ellipticum* extract were docked to the four target enzymes, Table 2. The compounds showed a considerable binding affinity and a good fit at the binding sites of the four enzymes, manifested by the scoring function values of their docking poses and the different interactions they afforded with the amino acid residues of these enzymes.

**Table 2:** Docking scores obtained about docking nine major compounds identified in *P. ellipticum* bark ethanolic extract.

**Compound name**	**Docking score (kcal/mol)**			
	**Elastase**	**Tyrosinase**	**Hyaluronidase**	**Collagenase**
Catechin pentoside	−21.32	−13.77	−14.50	−14.84
Coumaroyl glucose	−10.77	−13.19	−15.35	−12.75
Coumaroyl malic acid	−17.23	−21.00	−15.77	−19.28
Dihydroferulic acid glucoside	−13.19	−20.76	−13.91	−13.35
Dihydrosinapic acid glucoside	−18.08	−19.60	−14.64	−14.41
Ferulic acid glucuronide	−19.74	−19.39	−13.95	−15.51
Ethyl gallate	−13.15	−10.99	−11.38	−10.52
Shikimic acid malate	−16.49	−19.33	−11.91	−15.65
Sinapoyl malate	−14.11	−19.52	−13.63	−14.58
Quercetin	–	–	–	−15.37
Kojic acid	−13.32	−9.10	−7.99	–

**Fig. 2:**
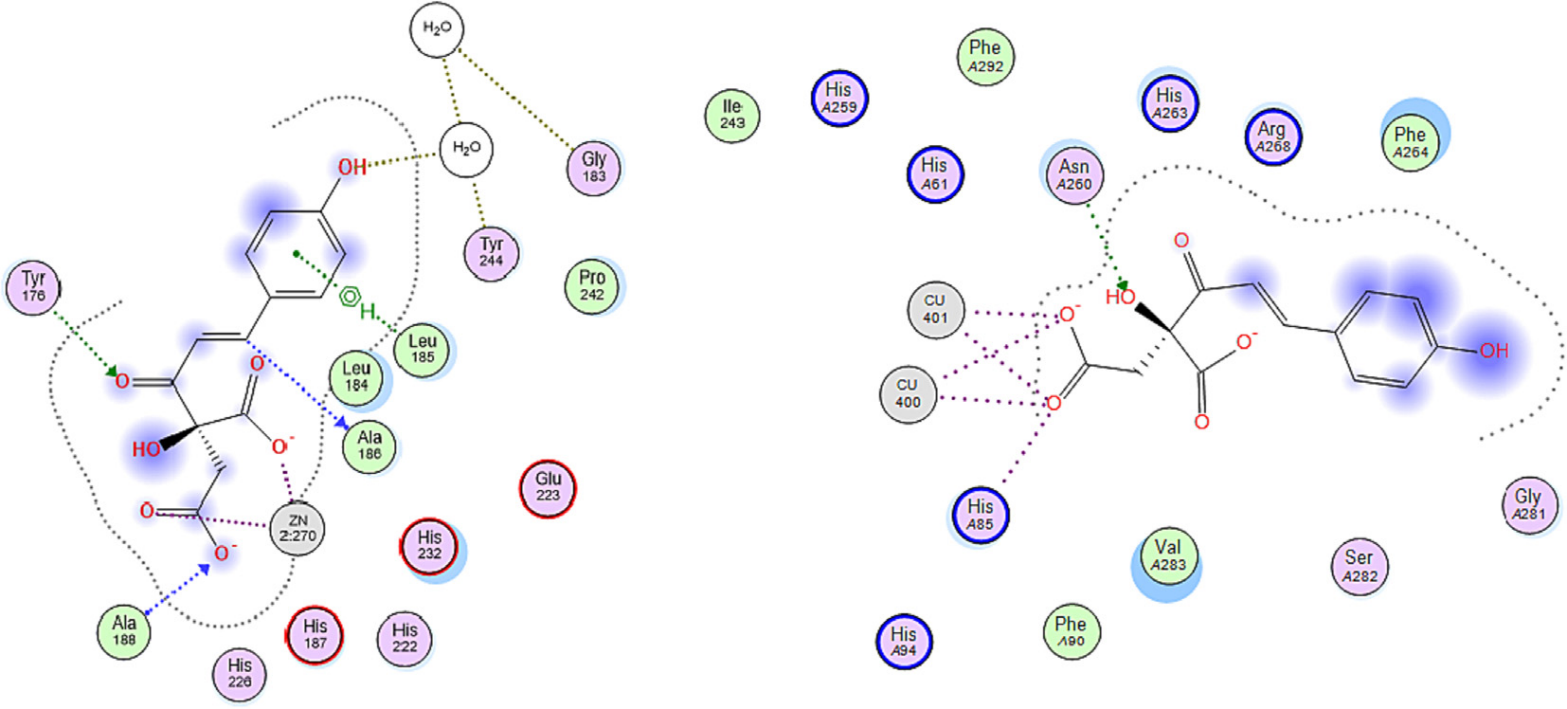
2D-docking poses of coumaroyl malic acid in the binding site of collagenase (left) and tyrosinase (right) enzymes.

The binding site of the metalloprotease collagenase, responsible for collagen degradation, is marked by the presence of Zn^2+^ ion and framed by the amino acids Leu185, Ala186, Ala188, Val219, His222, Glu223, Pro242, Tyr244, and Thr245. As shown in Table 2, six out of the nine compounds showed very comparable binding affinity relative to the reference inhibitor quercetin towards collagenase enzyme, where they had docking score values ranged between −19.28 and −14.41 versus −15.37 kcal/mol for quercetin. Coumaroyl glucose, dihydroferulic acid glucoside, and ethyl gallate showed lower affinity towards collagenase enzyme, which was reflected by their relatively higher docking scores of −12.75, −13.35, −10.52 kcal/mol, respectively. Interestingly, all the nine compounds showed ability to chelate the Zn^2+^ ion in the binding site, an interaction reported to be crucial for collagenase inhibitors (Kohno et al., 2006), which suggests high affinity of the extract’s compounds against collagenase enzyme. Coumaroyl malic acid showed the highest collagenase binding affinity (least docking score) with an estimated free binding energy of −19.28 kcal/mol. In addition to Zn^2+^ chelation (1.93 Å), this compound afforded hydrogen bonding interactions with Leu185 (3.81 Å) and Ala186 (3.25 Å), which are also afforded by the synthetic co-crystallized inhibitor, hydroxamic acid. The compound showed a hydrogen bond interaction as well with Ala188 (3.29 Å), Tyr176 (3.12 Å), and pi-H hydrophobic interaction with Leu185 (3.81 Å) at the binding site of collagenase enzyme, Fig. 2. The second and third top compounds with respect to the binding affinity towards collagenase enzyme were shikimic acid malate and ferulic acid glucuronide that showed docking scores of −15.65 and −15.51 kcal/mol, respectively. The two compounds afforded similar interaction types and distances showed by coumaroyl malic acid with the amino acids at the binding site of the enzyme. Noteworthy, the three compounds share the negatively charged carboxylate moiety that is able to chelate the zinc ion.

The crystal structure of tyrosinase shows that it is a metalloenzyme containing two tetragonal copper ions in its binding site that is framed by the amino acid residues Asn81, His85, Asn260, His263, Phe264, Arg268, Met280, Val283, and Glu322. Regarding interactions with the tyrosinase binding site residues, it is reported that Cu^2+^ ion chelation and the sigma-pi interaction with His263 are of utmost importance to tyrosinase inhibition (Ismaya et al., 2011, Elgamal et al., 2021). Viewing the docking results on tyrosinase enzyme reveals that all docked compounds showed better binding affinity than the reference inhibitor kojic acid where they showed docking scores between −21.00 and −10.99 versus −9.10 kcal/mol for kojic acid, Table 2. However, none of the compounds was able to afford both key interactions at any of their docking poses. Catechin pentoside and coumaroyl glucose did not chelate Cu^2+^ nor afford the hydrophobic interaction with His263 and showed relatively lower affinity to tyrosinase enzyme compared to the other compounds. Ethylgallate was not able to chelate Cu^2+^ but showed pi-pi interaction with His263, however it had the least estimated binding affinity (-10.99 kcal/mol) among all the nine compounds. The rest of the compounds were able to chelate Cu^2+^ ion and show some polar and non-polar interactions with the amino acid residues in the binding site but did not interact with His263. This could suggest low propensity of the extract to inhibit tyrosinase enzyme in view of the binding pattern of the compounds docked into this target enzyme. Again, coumaroyl malic acid showed the highest affinity upon docking to tyrosinase as it had the least minimum docking score of −21.00 kcal/mol, Table 2. It interacted with the Cu^2+^ ions via metal chelation (2.60 Å) and ionic (2.62 Å) interactions and afforded as well ionic interaction with His85 (3.01 Å) and hydrogen bond interaction with Asn260 (2.94 Å) residues in the binding site, Fig. 2. The second and third top compounds with respect to the binding affinity towards tyrosinase enzyme were dihydroferulic acid glucoside that revealed a docking score of −20.76 kcal/mol and polar interactions such as hydrogen bond interaction with Val283 (3.27 Å) and Cu^2+^ ions via metal chelation (2.45 Å) and ionic (2.50 Å) interactions and dihydrosinapic acid glucoside that had a docking score of −19.60 kcal/mol and afforded ionic interaction with His85 (2.97 Å), hydrogen bond interaction with Arg268 (3.06 Å), and interacted with Cu^2+^ ions as well via metal chelation (2.62 Å) and ionic (2.82 Å) interactions.

**Fig. 3:**
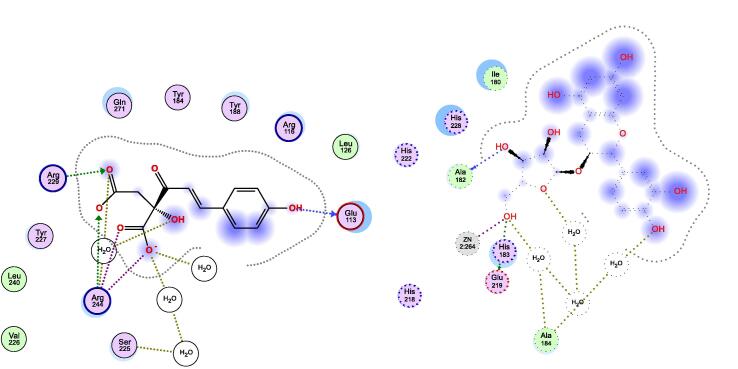
2D-docking poses of coumaroyl malic acid (left) and catechin pentoside (right) in the binding site of hyaluronidase and elastase enzymes, respectively.

The binding site of hyaluronidase enzyme is framed by several polar and non-polar amino acids including Tyr55, Asp111, Glu113, Arg116 Tyr184, Tyr227, Arg244, Gln271, Trp301, Ser303, and Ser304. The docking pose of the reference inhibitor kojic acid docked into hyaluronidase enzyme showed a score of −7.99 kcal/mol and iterated some of the amino acid interactions reported by hyaluronic acid such as the polar hydrogen bond interactions with Glu113, Tyr184, and Gln271 (Marković-Housley et al., 2000). All the extract’s compounds surpassed the reference kojic acid in terms of binding affinity towards hyaluronidase as their docking scores were in the range of −15.77 to −11.38 kcal/mol, which could suggest appreciable inhibitory potential of the extract against hyaluronidase enzyme. Interestingly, coumaroyl malic acid showed again the highest affinity towards hyaluronidase enzyme with a docking score of −15.77 kcal/mol and afforded different polar interactions with the amino acid residues in the enzyme’s binding site including ionic interaction with Arg244 (3.02 Å) and hydrogen bond interactions with Glu113 (2.95 Å), Arg229 (3.11 Å), and Arg244 (2.89 Å), Fig. 3. The second and third top compounds with respect to the binding affinity towards hyaluronidase enzyme were coumaroyl glucose and dihydrosinapic acid glucoside that iterated docking scores of −15.35 and −14.64 kcal/mol, respectively. Coumaroyl glucose showed hydrogen bond interactions with Tyr227 (2.94 Å) and Gln271 (2.77 Å), while dihydrosinapic acid glucoside afforded hydrogen bond interaction with Glu113 (2.78 Å) and two ionic bond interactions with Arg274 (2.88, 3.00 Å) in the binding site of hyaluronidase enzyme.

Like collagenase, elastase is a metalloprotease enzyme that degrades elastin in the ECM. The binding site is characterized by the presence of the Zn^2+^ ions and framed by the amino acids His172, Gly179, Ala182, His183, Ala184, Glu219, His222, His228, and Pro238. Regarding the potential of the extract’s compounds to bind to elastase enzyme, six compounds showed better docking scores (ranged between −21.32 to −10.77 kcal/mol) and hence better binding affinity towards the target enzyme, compared to the reference inhibitor kojic acid that had a score of −13.32 kcal/mol. They were able to chelate the Zn^2+^ ion and afford some of the reported interactions by elastase inhibitors with amino acids such as Ala182 and Glu219 (Bertini et al., 2005). The weakest affinity was shown by dihydroferulic acid glucoside, ethyl gallate, and coumaroyl glucose that had −13.19, −13.15, and −10.77 kcal/mol, respectively as docking scores, Table 2. However, catechin pentoside showed the highest affinity to the enzyme (-21.32 kcal/mol) and iterated the same interactions afforded by the co-crystallized synthetic inhibitor, acetohydroxamic acid, with the binding site residues, including Zn^2+^ chelation (2.11 Å), and the hydrogen bonding interactions with Ala182 (2.78 Å) and Glu219 (2.84 Å), Fig. 3. The second and third top compounds with respect to the binding affinity towards elastase enzyme were ferulic acid glucuronide and dihydrosinapic acid glucoside. The former had a docking score of −19.74 kcal/mol and interacted with the Zn^2+^ ion in the binding site via ionic (1.92 Å) interaction and afforded hydrogen bond interaction as well with Ala182 (2.92 Å) and two other indirect hydrogen bond interactions through water with Leu181 and Ala184. Dihydrosinapic acid glucoside, on the other hand, showed a docking score of −18.08 kcal/mol and afforded zinc chelation (2.25 Å), two hydrogen bond interactions with Glu219 (2.75 and 3.22 Å), ionic interaction with His172 (3.22 Å), and indirect hydrogen bond interactions with Leu181 and Ala184 in the binding site of the enzyme. The 3D docking poses of all the ligands bound to their respective enzyme proteins are included in Table S1 in the supplementary file.

#### 3.2.2 Molecular dynamics

Herein, 100 ns molecular dynamics simulations of ligands with the best docking poses for elastase, collagenase, hyaluronidase and tyrosinase enzymes were performed. The relationships between protein structures and ligands were determined by trajectory analysis (Hollingsworth & Dror, 2018). In the molecular dynamics simulation of the elastase protein structure, it was shown that the catechin pentoside remained in the protein structure where it was first docked during the simulation period. The main reason for this situation is the coordination of the oxygen at the hydroxyl end of the catechin pentoside with the zinc atom in the active site of elastase. The zinc atom coordinated the ligand to form the seesaw geometry. This geometry was maintained throughout the simulation and was identified as the strongest interaction linking the ligand to the protein structure. Catechin pentoside started its molecular dynamics simulation by positioning parallel to the structure formed by the seesaw geometry. After 7 ns, it underwent a conformational change, converging to residue His172. During this proximity, both aromatic rings in the ligand started to make pi-pi interactions with the His172 residue. The ligand and elastase protein structure maintained this conformation and interactions in the up to 77 ns. Afterwards, the ether bond turned around 180°away from His172 and one of the aromatic rings interacted with the His222 residue. Molecular dynamics simulation ended in this conformation for elastase.

The most important interaction in the molecular dynamic simulation of coumaroyl malic acid anchored to the collagenase protein structure is the octahedral coordination of its two carboxylic acid ends with the zinc atom in the active site. The residues Glu223, His232, and His226 are other components involved in coordination. This geometry was preserved throughout the simulation. With the aromatic ring at the other end of the ligand, the simulation started inside the active site framed by the residues Tyr244, Ile243, Gly182 and Leu184. However, it left this pocket 2 ns after the simulation started. The aromatic ring began to fluctuate in the solvent medium from this time on. The aromatic ring in the coumaroyl malic acid migrated to the region of Tyr176 and Pro177 residues after a few nanoseconds. The hydroxy group attached to the aromatic ring has started to form hydrogen bond with the residues in this region. In addition, the aromatic ring made a pi-pi interaction with the residue Tyr176. The aromatic ring group in the ligand retained these interactions up to 52 ns. It then abandoned these interactions and began to fluctuate in the solvent medium. When the simulation came to 90 ns, the aromatic ring group returned to its original place and was hydrogen bonded between the Pro177 and the hydroxyl end, and the simulation ended while these interactions continued. The ligand remained in the binding pocket throughout the whole simulation.

Coumaroyl malic acid in the hyaluronidase enzyme started molecular dynamics simulation with strong interactions with residues Arg244, Arg229 and Gln271 through the two carboxylic acid ends. These crucial interactions were hydrogen bonds and salt bridges that allowed the compound to maintain its position in the binding pocket up to 18 ns. Then, these interactions began to weaken but did not disappear and were maintained throughout the whole simulations. This compound found in the plant extract is the main compound responsible for inhibiting the hyaluronidase enzyme.

The coumaroyl malic acid in the tyrosinase enzyme started the simulation by forming coordination with the two copper ions via its carboxylic acid groups. This interaction lasted throughout the molecular dynamics simulation and fixed this end of the ligand to the protein structure. The other portion of the ligand (i.e., the portion of the aromatic ring) is positioned perpendicular to these interactions. These interactions and locations remained unchanged throughout the whole simulation.

##### 3.2.2.1 Root-mean-square deviation (RMSD) analysis

**Fig. 4:**
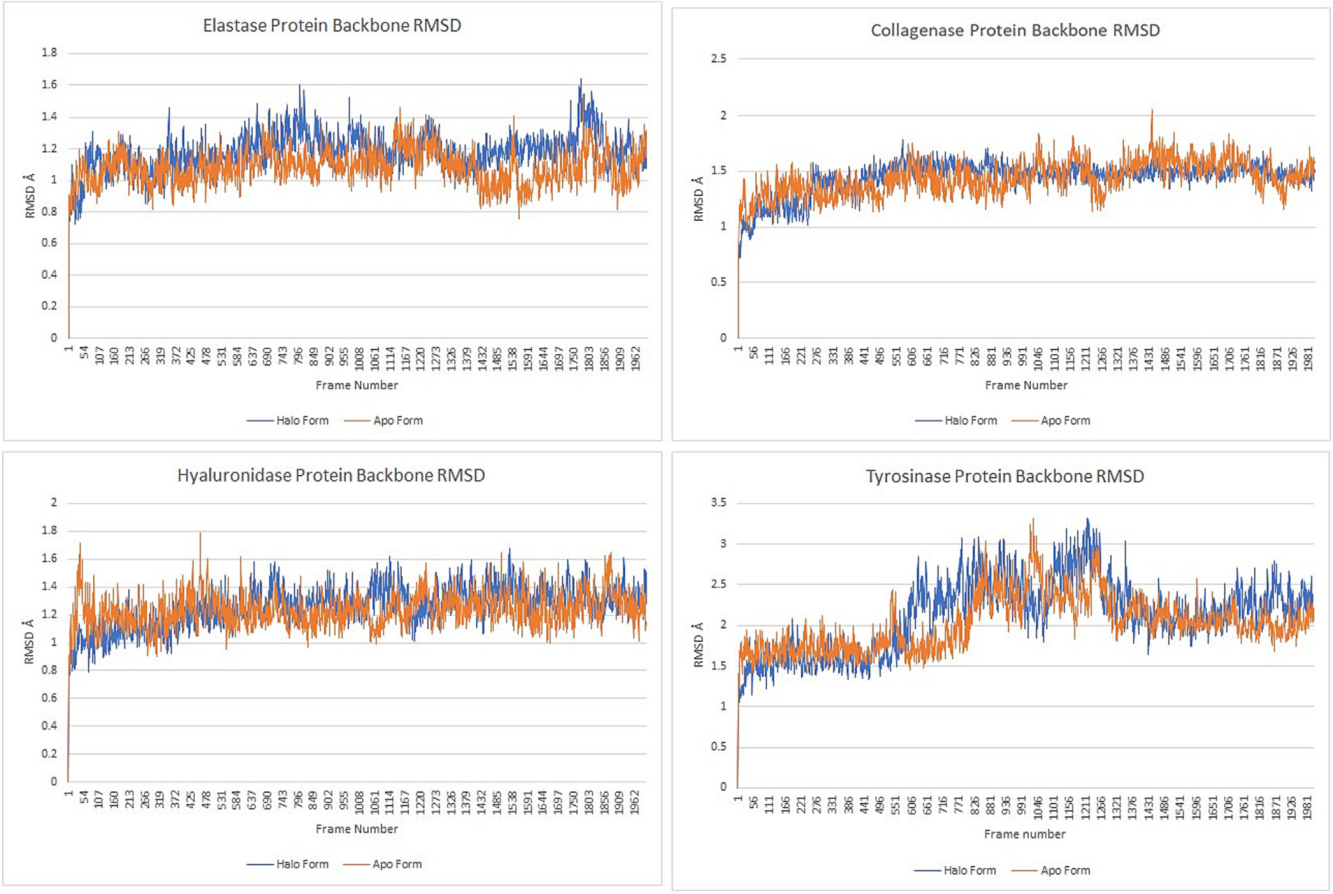
RMSD plots of elastase (catechin pentoside), collagenase (coumaroyl malic acid), hyaluronidase (coumaroyl malic acid) and tyrosinase (coumaroyl malic acid) proteins in molecular dynamics simulations.

RMSD plots are very useful to see and quantify the difference between the initial and final positions of both protein structure and ligand during molecular dynamics simulation. All protein frameworks are first aligned on the reference frame backbone, and then the RMSD is calculated based on atom selection. Changes in the protein RMSD value provide insights about the changing conformations of the protein structure throughout the simulation. Apo form RMSD and Halo form RMSD plots were superimposed to understand and compare how conformational changes of protein backbone change in the presence and absence of ligand, Fig. 4.

In the beginning of simulations, halo form RMSD values of elastase-catechin pentoside complex protein were largely similar to those of the Apo form. Later on, however, the halo form graph rose above the apo form graph to around 1.4 Å. This indicates that the catechin pentoside in the halo form causes a change in the conformation of the protein structure.

In the collagenase-coumaroyl malic acid complex, halo and apo forms were mostly progressed at the same values. However, while apo form RMSD values fluctuated more, this fluctuation was not observed in the halo form RMSD values.

The variability between the apo and halo forms of the hyaluronidase-coumaroyl malic acid complex was seen at the beginning of the simulation. As soon as the simulation started, the apo form graph increased sharply to an RMSD value of 1.4 Å. Then the graphs were separated from each other indicating strong and clear inhibition of the enzyme. Although the graphs overlapped with each other in the final stages of the simulation, the average of the RMSD values indicated strong inhibition.

Although both forms of the tyrosinase-coumaroyl malic acid complex started the simulation by obtaining the same RMSD values, the halo form RMSD values remained above the apo form ones, which indicates that a conformational change occurs in the protein structure when ligand binds to the tyrosinase enzyme. This change is consistent with the natural conformational change of the apo form, but more deviation from the mean position was observed, Fig. 4.

##### 3.2.2.2 Root-mean-square fluctuation (RMSF) analysis

**Fig. 5:**
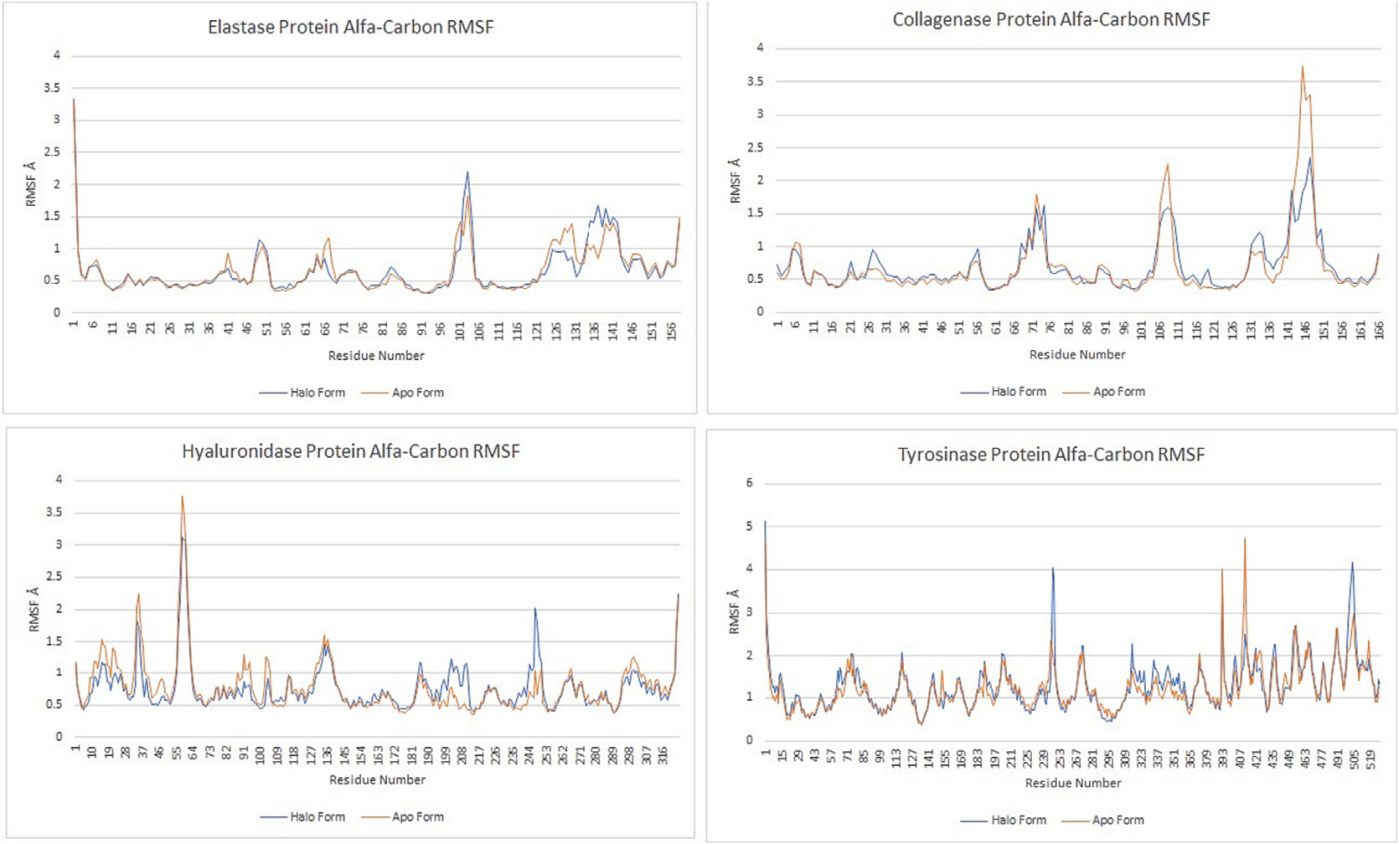
RMSF plots of elastase (catechin pentoside), collagenase (coumaroyl malic acid), hyaluronidase (coumaroyl malic acid) and tyrosinase (coumaroyl malic acid) proteins in molecular dynamics simulations.

RMSF plots were used to track local changes in protein structures throughout the molecular dynamic simulations. It was found that the free ends in the protein structures fluctuate a lot during the simulations. Halo and apo form plots were superimposed to examine the effects of ligands on enzyme protein structures, thus, the effects of ligands on local fluctuations could be studied, Fig. 5.

As for the elastase-catechin pentoside complex, it was found that there is a differentiation in the protein structure in two regions. This variability manifests itself as excessive fluctuation of the halo form at two points. That is, when the ligand is attached to the enzyme, certain regions in the protein structure fluctuate more than others. These regions are clearly visible in Fig. 5. Other parts of the protein structures showed substantially the same fluctuation characteristics.

In the collagenase-coumaroyl malic acid complex, it was observed that the graph of the halo form rose above that of the apo form at two points in the local fluctuations in the enzyme. This means that the compound caused less fluctuation in the two regions of the protein structure that fluctuated the most. In other regions of the protein structure, apo and halo form graphs were the same.

The halo and apo form graphs of the hyaluronidase-coumaroyl malic acid complex were mostly the same. However, it was found that there was little difference at some points and the halo form fluctuated more. These fluctuating parts constituted the middle parts of the protein structure. The fact that the ends fluctuate more than the apo form in the RMSF graphs, which is known to fluctuate more, pinpointed the local changes occurred when the coumaroyl malic acid was attached to the enzyme.

Fluctuations in the tyrosinase enzyme occurred in the middle parts and showed that the halo form of the tyrosinase-coumaroyl malic acid complex was more fluctuating. The last parts of the graph, which obviously fluctuated a lot, gave mixed signals. This is because this region forms the end parts of the protein structure.

##### 3.2.2.3 MM-GBSA analysis

**Fig. 6:**
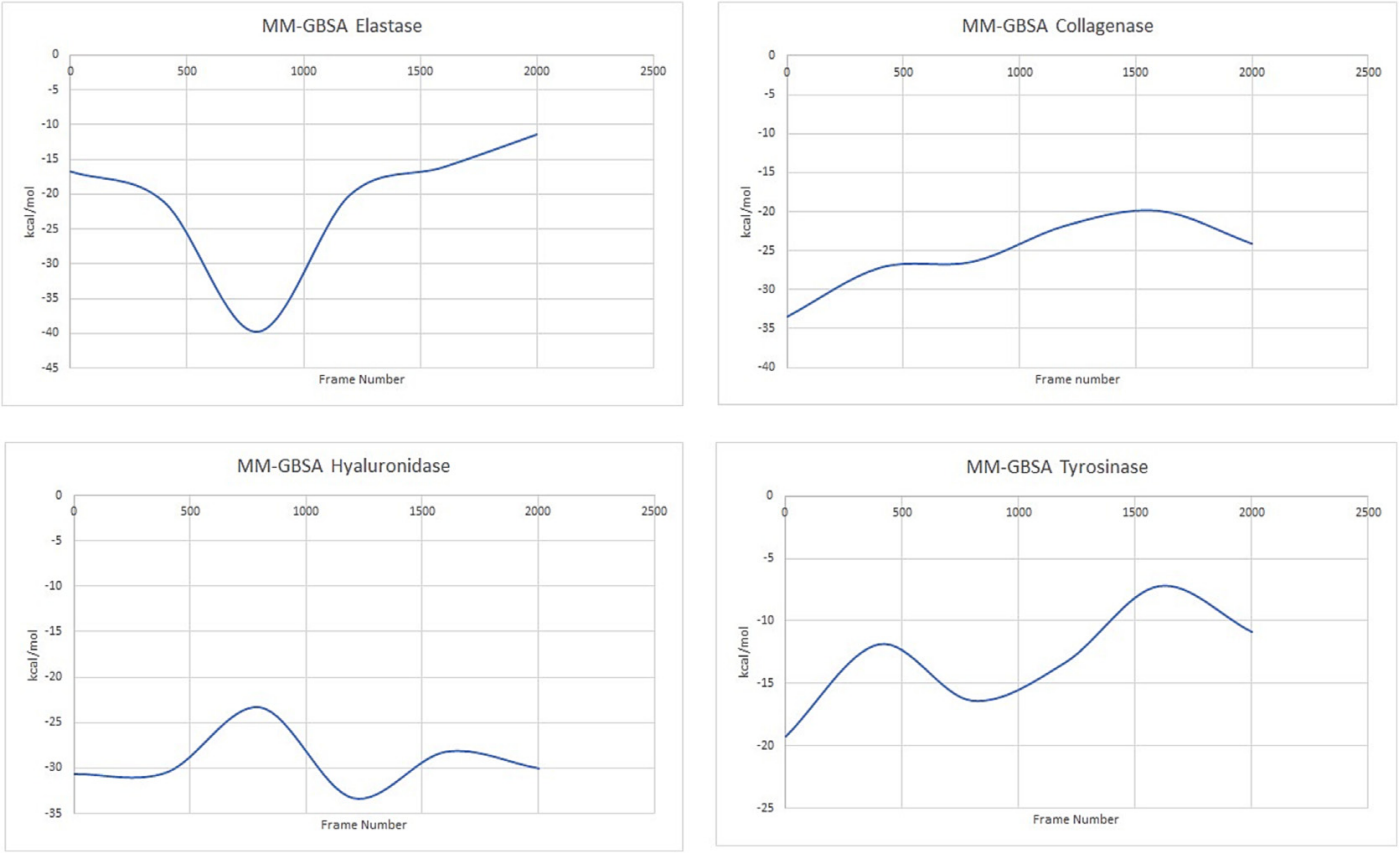
MM-GBSA plots of elastase (catechin pentoside), collagenase (coumaroyl malic acid), hyaluronidase (coumaroyl malic acid) and tyrosinase (coumaroyl malic acid) proteins in molecular dynamics simulations.

The MM-GBSA calculation was successfully performed every 400 frames for all the complexes in the different simulations, Fig. 6. The data were all negative values, which demonstrates the potential for ligands in all complexes to interact voluntarily with the protein structures of interest. In the MM-GBSA calculation of the catechin pentoside-elastase complex, the free energy decreased to −40 kcal/mol. The complex was most stable during this time period. Afterwards, the value in the graph increased and reached the value of −8 kcal/mol, which shows that the complex had a very stable structure. The complex formed by collagenase and coumaroyl malic acid showed an upward trend from –33 kcal/mol to −20 kcal/mol. However, after this value, it decreased rapidly to −25 kcal/mol. The rise in energy did not last long nor hit the positive indicating the potential of the complex to be quite stable. The complex formed by the hyaluronidase and coumaroyl malic acid fluctuated around the value of −30 kcal/mol but continued in a straight direction without showing any trend that could indicate the high potential stability of the formed complex. The tyrosinase-coumaroyl malic acid complex moved in an increasing trend but remained in the negative range throughout the whole simulation. This indicates that the complex had a high potential to be stable during the specified 100 ns time interval. When each of the complexes was examined separately, it was notable that the stability of these complexes is conferred by the interaction between the ligand and the metal in the respective protein’s active site. This highlights the importance of the metal’s charge density in potentiating the stability of the complex. All of the MM-GBSA calculations suggest that the studied compounds contribute largely to the enzyme inhibitory activities of the extract.

### 3.3 *In vitro* antioxidant assays

In this work, the *in vitro* antioxidant potential of both (aqueous and ethanol) extracts of *P. ellipticum* leaves and bark were evaluated using DPPH and FRAP assays, Table 3. TPC was quantified by Folin-Ciocalteu method, and it varied from 74.95 to 161.39 mg GA/g extract. In addition, TFC was quantified using FeCl_3_ and the results ranged from 4.24 to 22.43 mg quercetin/g extract, Table 3. Noticeably, the ethanol bark extract displayed conspicuous antioxidant activity, where it exhibited IC_50_ of 56.45 μg/mL in DPPH and 15.34 mM FeSO_4_ equivalent/mg sample in FRAP assay, related to EGCG, Table 3. Additionally, it exhibited the highest total phenolic and flavonoids contents that represented 160.02 mg GA/g of extract and 22.43 mg quercetin/g of extract, respectively.

**Table 3:** In vitro antioxidant activities: DPPH, FRAP, TPC and TFC for the aqueous and ethanolic extracts of *P. ellipticum* leaves and bark.

**Plant organ**	**Extract**	**DPPH**	**FRAP**	**TPC**	**TFC**
		IC_50_, µg/mL	mM of FeSO_4_/g extract	mg GA/g extract	mg QE/g extract
Leaf	Aqueous	44.12 ± 0.15	17.56 ± 0.51	150.71 ± 18.48	4.37 ± 2.23
	Ethanolic	65.34 ± 0.25	10.75 ± 0.07	161.39 ± 10.02	6.56 ± 0.02
Bark	Aqueous	> 100	9.42 ± 0.08	74.95 ± 3.53	4.24 ± 1.00
	Ethanolic	56.45 ± 0.26	15.34 ± 0.03	160.02 ± 23.66	22.43 ± 0.01
Reference compounds	Quercetin	0.23 ± 0.01	–	–	–
	BHT	4.21 ± 0.08	–	–	–

TPC: Total polyphenols content; TFC: Total flavonoids content.

### 3.4 *In vitro* anti-aging activities

The four extracts were evaluated for their *in vitro* inhibiting activity against four enzymes involved in aging, namely elastase, tyrosinase, collagenase and hyaluronidase, Table 4. Among the studied extracts, the ethanol bark extract revealed potential activity as illustrated by its strongest inhibition against collagenase with IC_50_ of 45.67 µg/mL, followed by elastase, hyaluronidase, and showed the least inhibition against tyrosinase, compared to the reference inhibitors kojic acid and quercetin. However, the other three extracts demonstrated IC_50_ above 100 µg/mL.

**Table 4:** In vitro enzyme inhibitory activities of aqueous and ethanolic extracts of *P. ellipticum* leaves and bark.

**Plant organ**	**Extract**	**Parameter**			
		**Elastase**	**Tyrosinase**	**Hyaluronidase**	**Collagenase**
			IC_50_, µg/mL		
Leaf	Aqueous	> 100			
	Ethanolic				
Bark	Aqueous				
	Ethanolic	50.01 ± 1.50	54.4 ± 1.78	52.67 ± 1.06	45.67 ± 1.01
Reference compound	Kojic acid	21.60 ± 0.9	9.00 ± 0.9	14.46 ± 0.6	–
	Quercetin	–	–	–	24.83 ± 1.8

### 3.5 Antibacterial activities

#### 3.5.1 Minimal inhibitory concentration

**Fig. 7:**
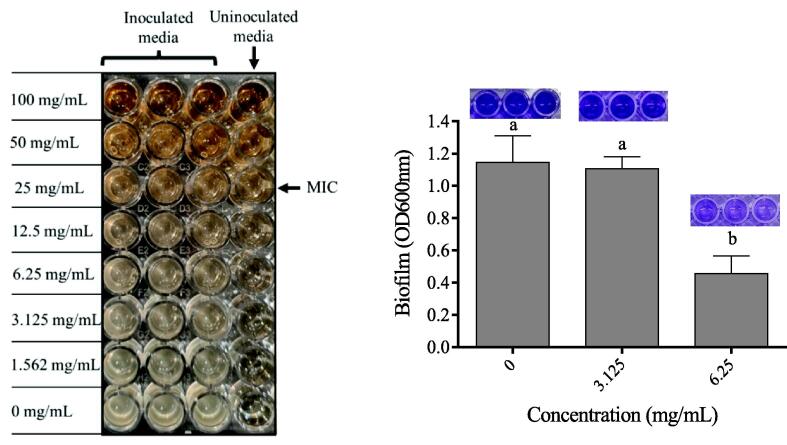
**(left)** The effect of the concentrations of the ethanolic extract of *P. ellipticum* bark on the growth rate of *P. aeruginosa*. **(right)** The effect of the extract at the doses of (0 mg/mL),3.125 mg/mL (1/8 MIC) and 6.25 mg/mL (1/4 MIC) on *P. aeruginosa* biofilm production. Letters in superscript indicate the statistical difference at *p* < 0.05.

The bacteriostatic effect of the *P. ellipticum* ethanol bark extract against *P. aeruginosa* was obtained at 25 mg/mL. Moreover, the bacterial growth rates in media containing the sub-MICs 1/4 (6.25 mg/mL) and 1/8 (3.125 mg/mL) were statistically not significant, Fig. 7 **(left)**. However, when the bacterium was treated with 1/4 MIC (6.25 mg/mL), a decrease in biofilm production of 60% was observed compared to the control, in which the extract was absent. In contrast, no significant variations in biofilm content were observed using 1/8 MIC (3.125 mg/mL) relative to the control media, Fig. 7 **(right)**.

#### 3.5.2 Effect of P. ellipticum extract on the swimming and swarming mobilities

**Fig. 8:**
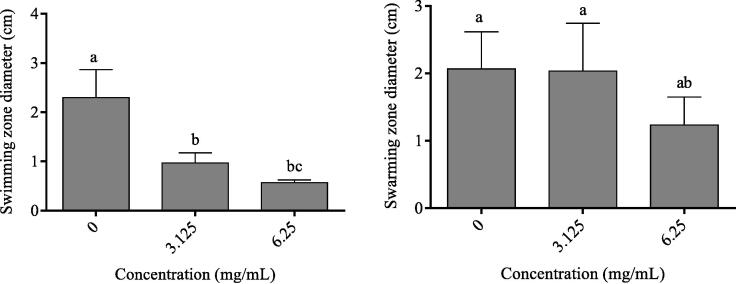
Effect of sub-MICs of *P. ellipticum* bark ethanolic extract on the swimming **(left)** and swarming **(right)** motilities of *P. aeruginosa* on plates. Letters in superscript indicate the statistical difference at the 0.05 level.

The mobilities of *P. aeruginosa* monitored on plates revealed that compared to media without extract (0 mg/mL), *P. ellipticum* ethanol bark extract significantly impaired the swimming mobility by 58.26 and 75.65 % using 1/8 (3.125 mg/mL) and 1/4 (6.25 mg/mL) MICs, respectively, Fig. 8. As for the swarming motility, even the 1/4 MIC reduced the swarming diameter by up to 40.29 %. No significant decrease was noticed compared to the control as well as to media with 1/8 MIC, Fig. 8.

## 4 Discussion

Cumulative oxidative stress accompanied with formation of unstable free radicals such as ROS in the skin cells is a major mechanism of skin aging as these species can cause lipid peroxidation, protein damage and mutation of DNA resulting in many structural and functional changes in the skin that in turn initiates many pathological conditions and ailments. The skin has its own antioxidant defense mechanisms, in which enzymes and other antioxidants can react with or scavenge ROS, thus keeping them from reacting with the different biological targets within the cells. Indeed, antioxidant substances show significant ability to prevent and/or manage different skin diseases and aging. During the last decade, several phytochemicals as polyphenolics with substantial antioxidant activities have been investigated to prevent or retard skin aging (Petruk et al., 2018). Thus, it is highly demanded to search for and investigate the antioxidant and antiaging properties of different medicinal plants so that it could be used for managing and retarding skin aging process. In this context, the present work focused on the application of LC-MS analysis to annotate the phytochemical profiling of the ethanol bark extract of *P. ellipticum*, adopting in silico molecular docking and molecular dynamic studies to estimate the binding affinity of the major extract’s compounds against the four enzymes involved in the aging process, the *in vitro* antioxidant and antiaging activities of the aqueous and ethanol extracts of *P. ellipticum* leaves and bark, together with evaluating its antibacterial and antibiofilm activities.

Among the four examined extracts, the ethanol bark extract had the greatest TPC and TFC besides its high antioxidant activity. The aerial parts of this plant from Mexico showed similar results (Ruiz-Terán et al., 2008). The phytochemical profile of this extract was determined through the LC-MS/MS analysis and revealed that the extract comprised high level of flavonoids, phenolic acids, their derivatives and procyanidins, indicating that the potential antioxidant activity of the extract is most likely because of these constituents. Flavonoids and phenolic acids are the most predominating classes of phytoconstituents in plants. They are powerful antioxidants that can protect the skin against penetration by radiation, and minimize inflammation and oxidative stress, therefore impact several pathways to protect against skin damage and aging. Their antioxidant activity is most probably due to their capability of scavenging unstable free radicals as ROS and RNS, decreasing the formation of ROS/RNS through inhibition of some enzymes or chelation of trace metals which are important for free radical formation, besides their ability to upregulate and enhance the antioxidant defense mechanisms (Kaurinovic and Vastag 2019). The antioxidant activity of the procyanidins (monomer, dimers, and trimers)-rich plant extracts and the formerly described antioxidant activities of quercetin and kaempferol glycosides beneficially verify their high contribution to the antioxidant potential of the corresponding extracts (Lesjak et al., 2018, Tawfeek et al., 2019, Silva dos Santos et al., 2021).

Collagen and elastin are important components in the extracellular matrix, high level of them is needed to maintain the skin strength, elasticity and flexibility. These two important biomolecules are rapidly degraded during aging due to the overproduction of collagenase (MMP13) and elastase (MMP12), respectively, which are metalloproteinases that enhance the breakdown of the peptide bonds in collagen and elastin. Also, elastase can stimulate other metalloproteinase enzymes, which enhance the matrix proteolytic degradation (Jabłońska-Trypuć et al., 2016). As soon as wrinkles begin to form, elastase is secreted by fibroblasts resulting into a decrease in skin elasticity and an elevation in elastic fibers tortuosity (Imokawa and Ishida 2015). Hence, blocking MMP13 and MMP12 would enhance skin elasticity and protect against skin aging. Tyrosinase (polyphenol oxidase), is a metalloenzyme that regulates the melanin production by the melanocytes, where it activates the hydroxylation of tyrosine into3, 4-dihydroxyphenylalanine (DOPA), then the oxidation of DOPA to give DOPA quinone (melanin precursor). Overproduction of melanin cause hyperpigmentation, which is a major symptom of skin aging and can be increased by many factors as disturbances in hormonal level, UV radiation and chemicals, therefore tyrosinase inhibitors can enhance skin appearance and lightening (Bae-Harboe and Park 2012). Hyaluronic acid (glycosaminogly derivative) is one of the essential components of the extracellular matrix and it has an important role in improving skin moisture, elasticity, tissues renewal, and repair. Hyaluronidase enzyme is one of hyaluronoglucosidase enzymes,controls the degradation of Hyaluronic acid at the bond between the N-acetylglucosamine and glucoronate units (Buhren et al., 2016), thus it is considered an important target for anti-aging a drugs. In this study, nine major secondary metabolites identified in the extract were docked to the four target enzymes, Table 2, whereas the rest of the compounds were previously characterized in *Euphorbia retusa* leaf extract and docked to the same target enzymes (Elgamal et al., 2021). Indeed, our current and previous *in silico* studies showed appreciable estimated free binding energies for the ligand–protein complexes obtained upon docking, which reflects high binding affinity of the extract’s compounds towards the different target enzymes playing key roles in the aging process, where the docked compounds showed<5 Å distance of the interactions between the docked compounds and the target amino acid residues in the binding sites of the corresponding enzymes that confirms their well fitness in these sites. The current study was able to highlight the strong inhibitory potential of three top ranking compounds that showed cross-binding affinity towards more than one target enzyme. Coumaroyl malic acid showed the best binding affinity towards collagenase, tyrosinase, and hyaluronidase enzymes; dihydrosinapic acid glucoside was among the top-ranking compounds towards tyrosinase, hyaluronidase, and elastase enzymes, while ferulic acid glucuronide was among the top-ranking compounds towards collagenase and elastase enzymes. Further bioassays and *in vitro* studies are highly recommended to confirm the antiaging activity of these compounds.

In this work we also confirmed the extract’s potential to inhibit the skin aging enzymes in an *in vitro* enzyme inhibition assay. The ethanol bark extract of *P. ellipticum* was turned out to be a rich source for antioxidant compounds, Table 1, which can act synergistically against target enzymes involved in skin aging process. For example, catechin, procyanidin, quercetin, kaempferol, myricetin derivatives, isorhamnetin have been found to have a good anti-elastase and anti- collagenase activities (Hussin et al., 2019). Similar results were also obtained from other investigations on plants containing chlorogenic acid, catechins, quercetin glucoside, kaempferol rutinoside and isorhamnetin rutinoside, which exhibited promising *in vitro* antiaging activity by interfering with these target enzymes (Elgamal et al., 2021, Szewczyk et al., 2021, Abdelfattah et al., 2022). As well, rutin, and quercetin 3-*O*-glucoside were tested for hyaluronidase inhibition and found to exhibit a strong inhibitory potential against the enzyme through various hydrogen bonding interactions with the amino acid residues at the binding site (M. Mohamed et al., 2020). Noteworthy, our molecular docking study has suggested appreciable inhibitory potential of the extract towards collagenase, elastase, and hyaluronidase enzymes and lower propensity to inhibit tyrosinase enzyme. This came in agreement with the *in vitro* results, which revealed that the extract had the lowest potency towards tyrosinase enzyme.

In this work, we also aimed to explore the antibacterial potential of the ethanol bark extract from *P. ellipticum* so that we could have integral insights about the possibility of developing new plant-based derma-cosmeceutical agents. *Pseudomonas aeruginosa* is a Gram-negative bacillus and most associated with opportunistic infections, which range from superficial to deep skin infections. *P. aeruginosa* is characterized by its ability to acquire resistance against antibacterial agents, therefore the proper application of anti-pseudomonal treatment is highly important. Its resistance is most likely due to biofilm production, where the bacteria are clustered and enclosed in their extracellular matrix. The vast majority of the current antibacterial agents can decrease the number of bacteria in biofilms, without complete elimination of the biofilms, owing to their antibiotic resistance, leading to infection recurrence (Mulcahy et al., 2014, Ciofu and Tolker-Nielsen, 2019). Herein, we evaluated the anti-biofilm potential of the ethanol bark extract of *P. ellipticum* and its impact on swimming and swarming mobilities of *P. aeruginosa* by the two concentrations (1/4 and 1/8 MICs). The dose 6.25 mg/mL (1/4 MIC) significantly inhibited the biofilm production and reduced the swimming and swarming mobilities of *P. aeruginosa.* Similar results were illustrated previously for procyanidin containing extracts such as *Salix tetrasperma* bark and flower (Mostafa et al., 2020), *Ximenia americana* leaf (Bakrim et al., 2022).

## 5 Conclusion

Plants are considered potential sources of natural bioactive secondary metabolites. Totally, in this work, 35 secondary metabolites comprising proanthocyandins, flavonoids, phenolic acids, and their derivatives, were tentatively identified in ethanol bark extract of *P. ellipticum*. Our study also showcased the importance and the substantial potential of the natural products, especially polyphenols, to serve as antioxidants, antibacterial, and competitive inhibitors of target key enzymes (collagenase, elastase, tyrosinase, and hyaluronidase) in skin aging, thus improving the overall skin health. These findings may assist in the application of *P. ellipticum* bark extract in future in cosmetics and management of skin aging.

## Author contributions

Conceptualization, M.F.M. and M.S.; Investigation, E.F., I.M., N.T., M.A.O. and W.B.B.; software, A.B.O., M.AO.A. and K.W.O. ;  Validation, M.S.; Writing – original draft, E.F., I.M., N.T., M.A.O. and W.B.B.; Writing – review & editing, M.F.M and M.S.

## Funding

This research did not receive any specific grant from funding agencies in the public, commercial, or not-for-profit sectors.

## Declaration of Competing Interest

The authors declare that they have no known competing financial interests or personal relationships that could have appeared to influence the work reported in this paper.
