# Supplementary material for: Dermato-cosmeceutical properties of Pseudobombax ellipticum (Kunth) Dugand: Chemical profiling, in vitro and in silico studies
Source: Saudi Pharm J. 2023 Sep 1;31(10):101778. doi: 10.1016/j.jsps.2023.101778 (PMC10511495; doi:10.1016/j.jsps.2023.101778)
Supplement: Supplementary data 1 [file mmc1.docx]

**Dermato-cosmeceutical properties of *Pseudobombax ellipticum* (Kunth) Dugand: Chemical profiling, *in vitro* and *in silico* studies**

Methods

*Molecular Docking*

The molecular docking was done using the default protocol of the molecular operating environment software (MOE2022.v11.18.1) to perform the pre-docking preparations, the docking runs, and to visualize the potential poses and interactions. The enzymes’ structures were downloaded from protein data bank (www.pdb.org) with the corresponding bound ligands. The pdb codes were elastase (PDB id: 1Y93), tyrosinase (PDB id: 2Y9X), hyaluronidase (PDB id: 1FCV), and collagenase (PDB id: 2D1N). The proteins’ 3D structures were prepared using the structure preparation Quickprep panel in MOE. This allows protonation of structures, deletes the unbound water molecules, corrects the structural errors and affords energy minimization to 0.0001 gradient. The compounds were downloaded from PubChem as (.sdf) files then compiled into one database. The respective co-crystallized and reference ligands for each enzyme were inserted to the database for the purpose of docking protocol validation. Self-docking of these ligands to each of the corresponding enzymes retained RMSD values below 2Å between the docked pose and X-ray crystal structure pose, which is usually acceptable in the molecular docking procedure (Alqahtani et al., 2019; Muteeb et al., 2022). All ligands were prepared by the database wash tool to adjust formal charges for strong acids and bases and to adjust bond lengths’ scales. Ligands were also checked for any structural bonding errors and were then adjusted to the most abundant tautomeric state from the protomers panel and atomic partial charges were set. The binding site is identified according to the default protocol of the software to be within 5 Å from the co-crystallized ligand. The docking was implemented using triangle matcher as a placement method, refinement was done using induced fit. Scoring of the potential docking solutions was done using the default London dG scoring function and the refined poses were scored using the default GBVI/WSA dG scoring function. The output files were explored, and the resulting poses were filtered according to their scores in addition to the interactions they exhibited with the amino acids of the binding site.

As for the docking scores, the generated poses were first scored using London dG scoring function, refinement of the poses was implemented by induced fit to allow for ligand flexibility, the final energy was evaluated using the Generalized Born solvation model (GB/VI) which is a forcefield-based scoring function that estimates the free energy of binding of the ligand (Elgamal, El Raey et al. 2021).

**Table S1**: 3D docking poses of *P. ellipticum* extract’s selected compounds upon docking into enzymes involved in skin aging.

| **Compound** | **3D docking pose** |
| --- | --- |
| **Collagenase** |  |
| Catechin pentoside | 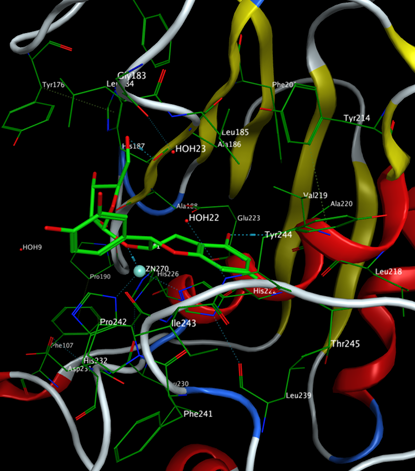 |
| Coumaroyl glucose | 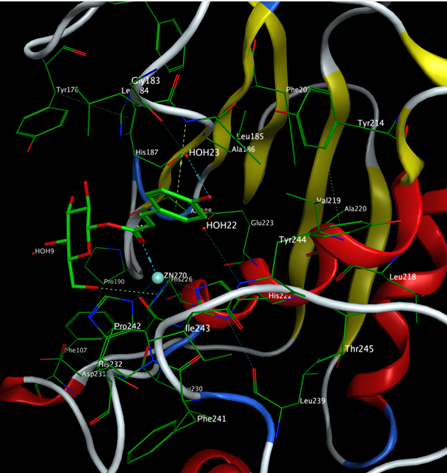 |
| Coumaroyl malic acid | 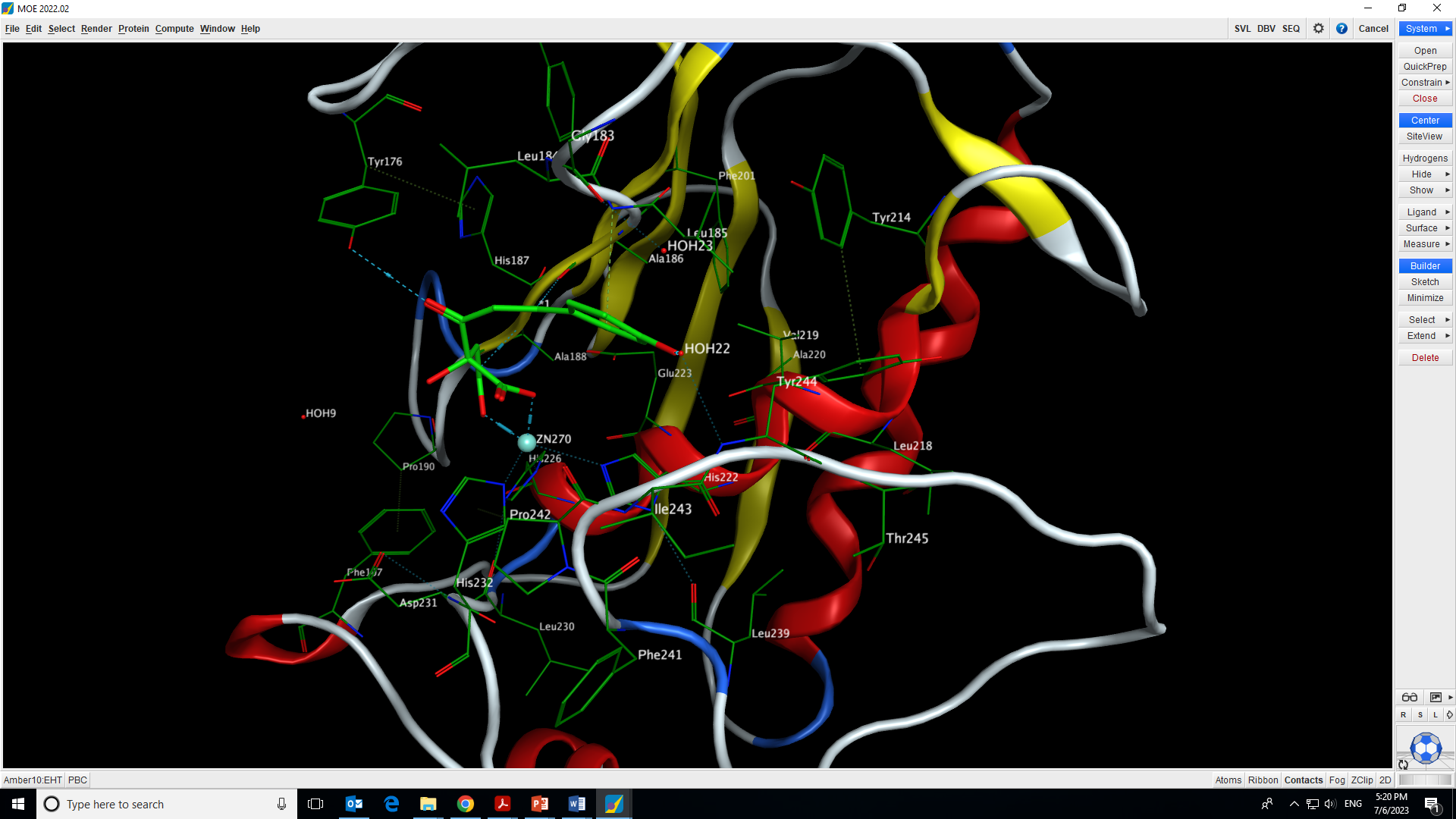 |
| Ferulic acid glucuronide | 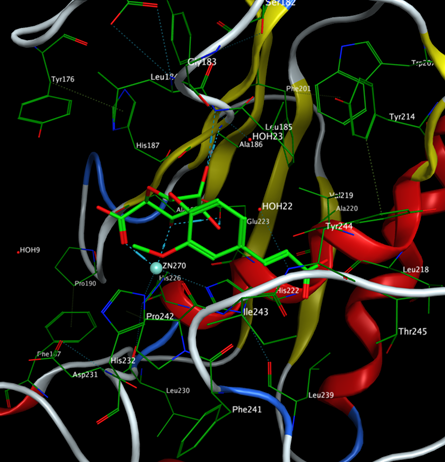 |
| Ethyl gallate | 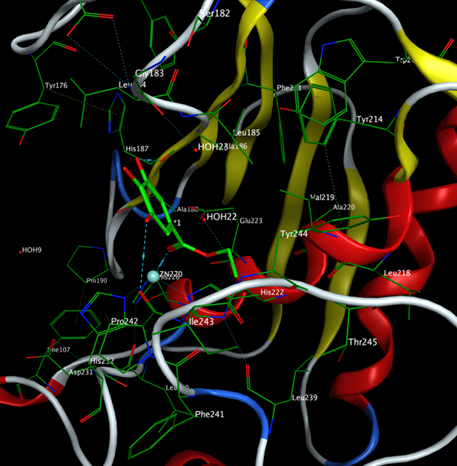 |
| Shikimic acid malate | 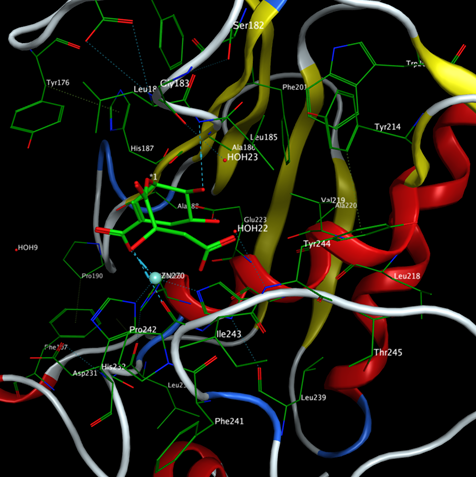 |
| Sinapoyl malate | 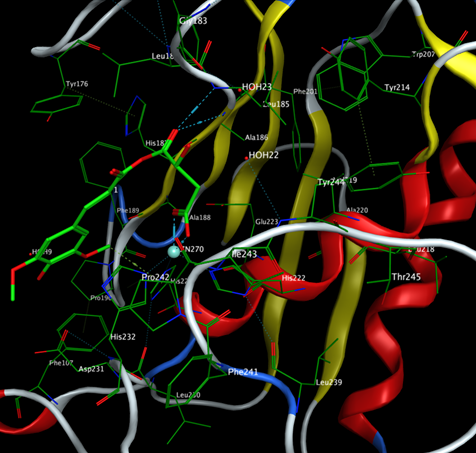 |
| **Tyrosinase** |  |
| Catechin pentoside | 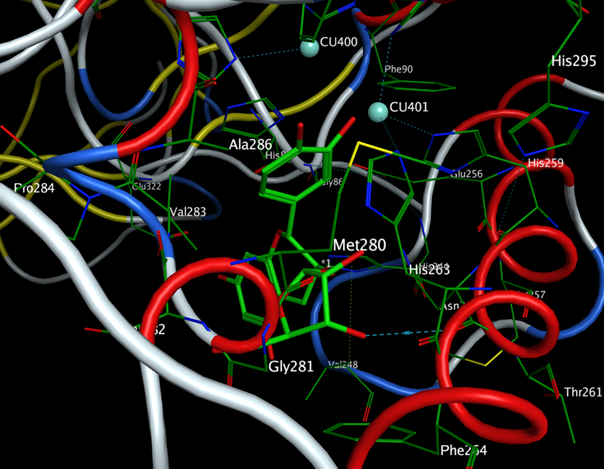 |
| Coumaroyl glucose | 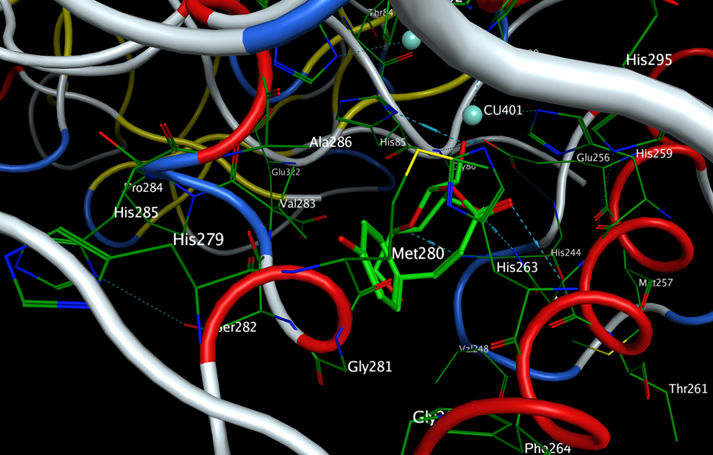 |
| Coumaroyl malic acid | 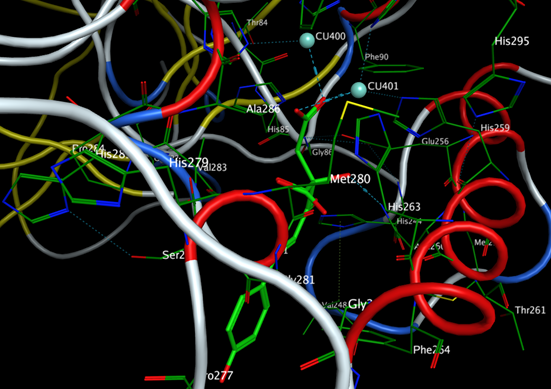 |
| Ferulic acid glucuronide | 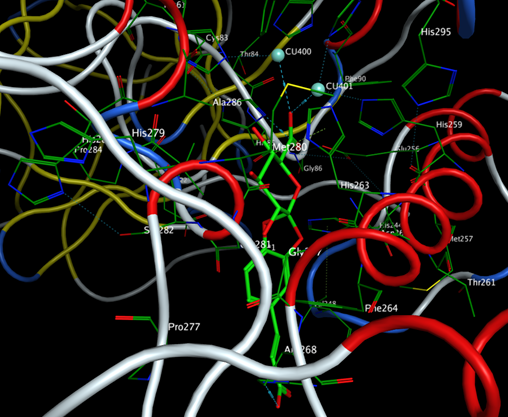 |
| Ethyl gallate | 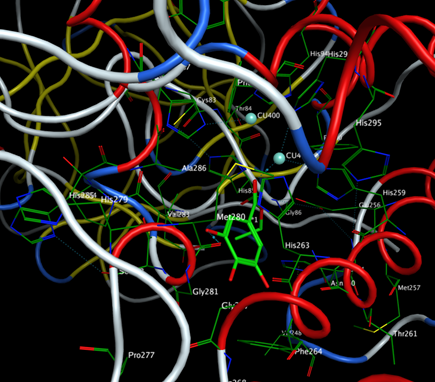 |
| Shikimic acid malate | 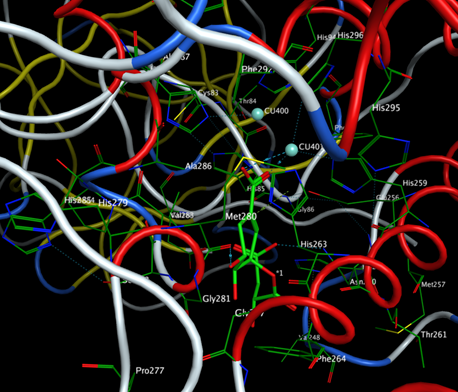 |
| Sinapoyl malate | 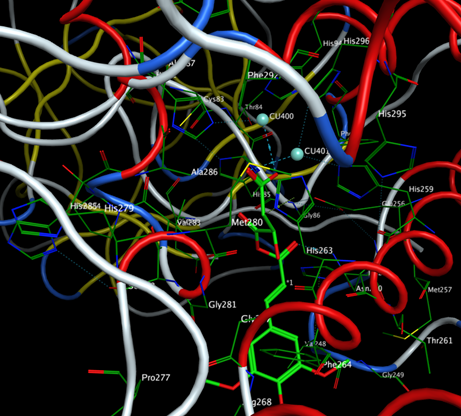 |
| **Hyaluronidase** |  |
| Catechin pentoside | 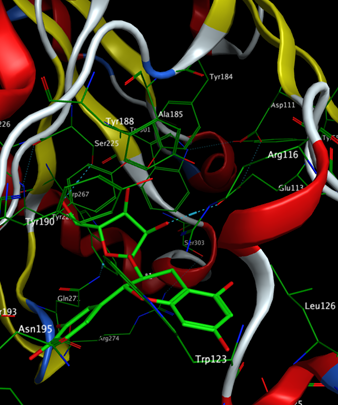 |
| Coumaroyl glucose | 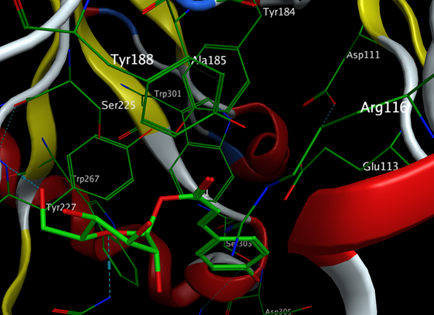 |
| Coumaroyl malic acid | 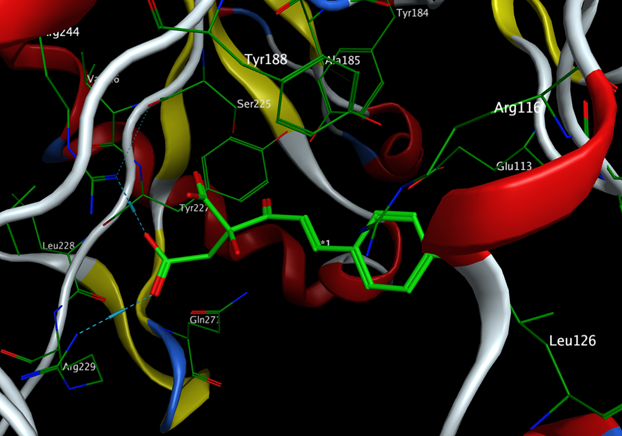 |
| Ferulic acid glucuronide | 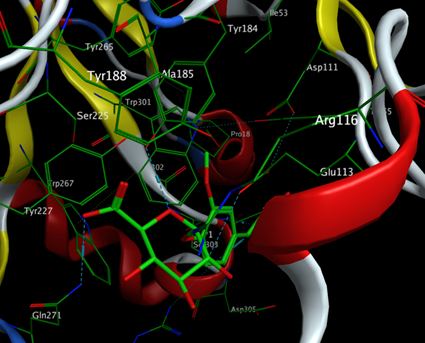 |
| Ethyl gallate | 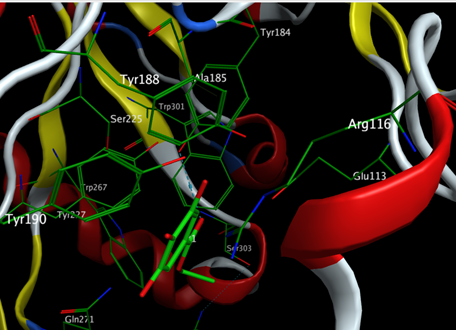 |
| Shikimic acid malate | 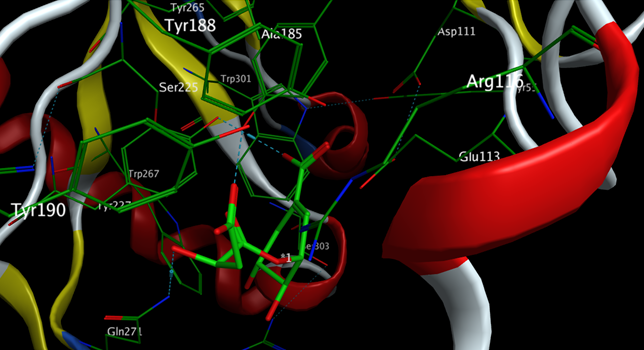 |
| Sinapoyl malate | 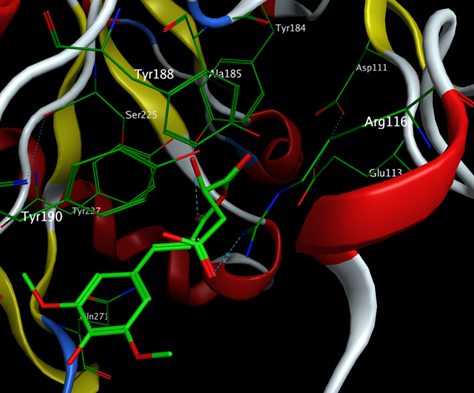 |
| **Elastase** |  |
| Catechin pentoside | 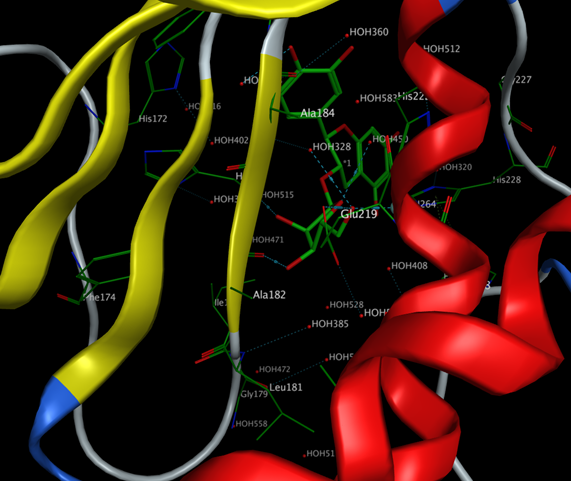 |
| Coumaroyl glucose | 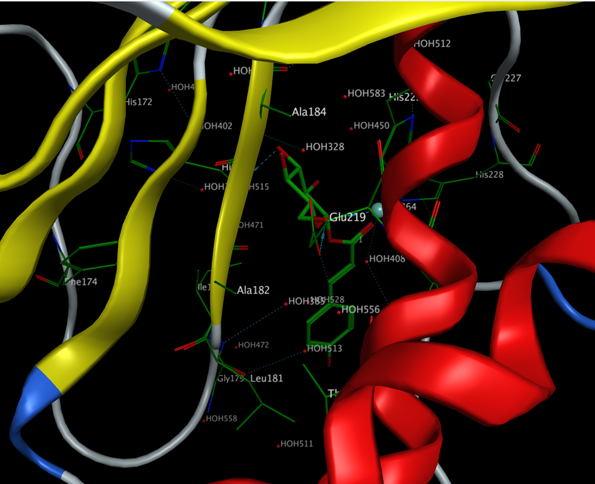 |
| Coumaroyl malic acid | 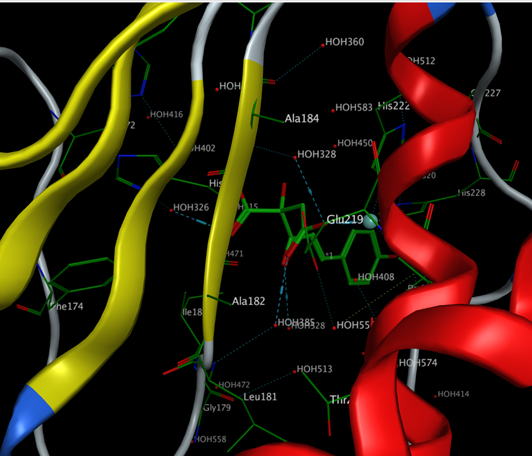 |
| Ferulic acid glucuronide | 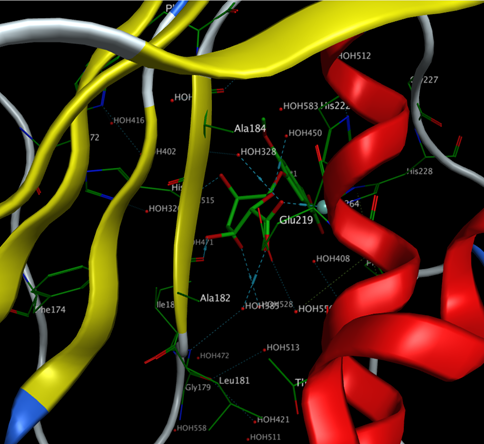 |
| Ethyl gallate | 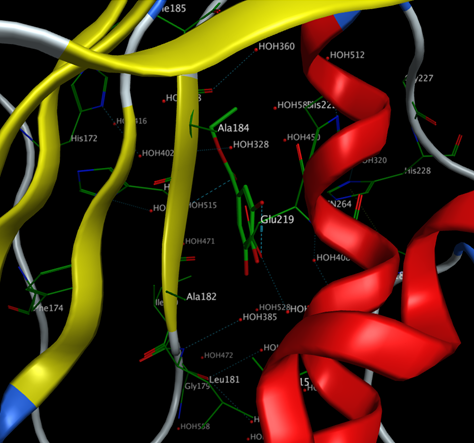 |
| Shikimic acid malate | 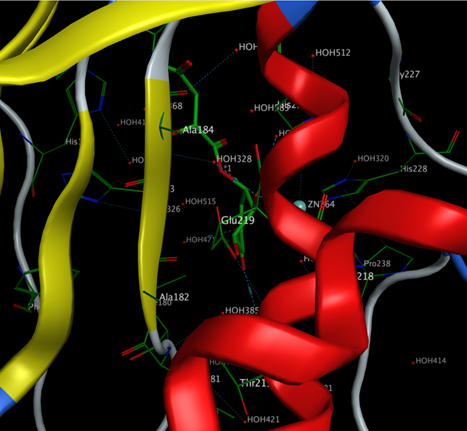 |
| Sinapoyl malate | 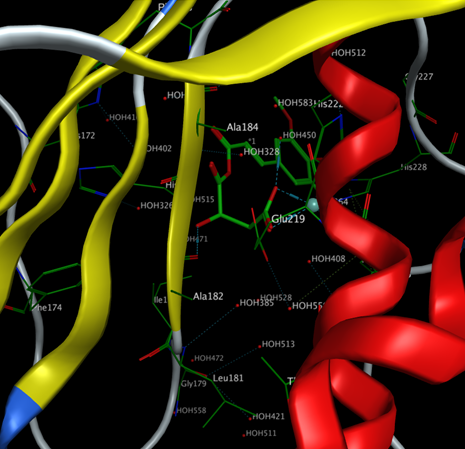 |

**REFERENCES**

Elgamal, A. M., M. A. El Raey, A. Gaara, M. A. Abdelfattah and M. Sobeh (2021). "Phytochemical profiling and anti-aging activities of Euphorbia retusa extract: in silico and in vitro studies." Arabian Journal of Chemistry **14**(6): 103159.
